# Design and selection of anti-PD-L1 single-domain antibody and tumor necrosis factor superfamily ligands for an optimal vectorization in an oncolytic virus

**DOI:** 10.3389/fbioe.2023.1247802

**Published:** 2023-11-15

**Authors:** Christelle Remy, Elodie Pintado, Marshall Dunlop, Shirley Schön, Patricia Kleinpeter, Homa Rozanes, Laetitia Fend, Renée Brandely, Michel Geist, Delphine Suhner, Eline Winter, Nathalie Silvestre, Claire Huguet, Peter Fitzgerald, Eric Quéméneur, Jean-Baptiste Marchand

**Affiliations:** ^1^ Transgene SA, Strasbourg, France; ^2^ Randox Laboratories Ltd., Crumlin, United Kingdom

**Keywords:** oncolytic virotherapy, vaccinia virus, single-domain antibody, surfactant protein D, PDL1, TNFSF, CD40, 4-1BB

## Abstract

Arming oncolytic viruses with transgenes encoding immunomodulators improves their therapeutic efficacy by enhancing and/or sustaining the innate and adaptive anti-tumoral immune responses. We report here the isolation, selection, and vectorization of a blocking anti-human PDL1 single-domain antibody (sdAb) isolated from PDL1-immunized alpacas. Several formats of this sdAb were vectorized into the vaccinia virus (VV) and evaluated for their programmed cell death protein 1 (PD1)/PD1 ligand (PDL1) blocking activity in the culture medium of tumor cells infected *in vitro*. In those conditions, VV-encoded homodimeric sdAb generated superior PDL1 blocking activity compared to a benchmark virus encoding full-length avelumab. The sdAb was further used to design simple, secreted, and small tumor necrosis factor superfamily (TNFSF) fusions with the ability to engage their cognate receptors (TNFRSF) only in the presence of PDL1-positive cells. Finally, PDL1-independent alternatives of TNFRSF agonists were also constructed by fusing different variants of surfactant protein-D (SP-D) oligomerization domains with TNFSF ectodomains. An optimal SP-D–CD40L fusion with an SP-D collagen domain reduced by 80% was identified by screening with a transfection/infection method where poxvirus transfer plasmids and vaccinia virus were successively introduced into the same cell. However, once vectorized in VV, this construct had a much lower CD40 agonist activity compared to the SP-D–CD40L construct, which is completely devoid of the collagen domain that was finally selected. This latest result highlights the importance of working with recombinant viruses early in the payload selection process. Altogether, these results bring several complementary solutions to arm oncolytic vectors with powerful immunomodulators to improve their immune-based anti-tumoral activity.

## Introduction

Oncolytic viruses (OVs) are well recognized as part of the preclinical and clinical immunotherapeutic arsenal to fight cancers (Wan et al., 2021). OVs encompass different classes of viruses that have in common a propensity to infect and/or replicate preferentially into tumor cells, leading to their destruction. For instance, the Copenhagen vaccinia virus (VV) deleted of both thymidine kinase (TK) and ribonucleotide reductase (RR) (Foloppe et al., 2019), was used as a backbone to generate all the recombinant OVs presented in this article. This double deletion of genes coding for enzymes involved in the metabolism of nucleotides restricts the virus replication to cells containing high levels of nucleotides (e.g., cancer cells) while sparing cells with low levels of nucleotides (e.g., resting cells). In addition to direct tumor cell lysis or immunological cell death caused by virus replication, tumor treatment by OV triggers massive infiltration by immune cells and enhances the adaptive anti-tumoral immune response that ultimately drive tumor regression (Fend et al., 2017). Finally, a few OVs with a large genome, such as VV, can integrate large pieces of foreign DNA, potentially encoding several transgenes that are expressed during the replication of the virus. Those transgenes may act synergistically with oncolysis and tumor immune infiltration to aid tumor regression, especially if they encode immunomodulators that boost the anti-tumoral adaptive immune response (Kleinpeter et al., 2016). Among the large list of known immunomodulators are some costimulatory molecules of the tumor necrosis factor superfamily (TNFSF) involved in both innate and adaptive anti-tumoral immune responses (Muller, 2023). For instance, both the CD40/CD40 ligand (also known as CD154/CD154L or TNFRSF5/TNFSF5) and the 4-1BB/4-1BB ligand (also known as CD137/CD137L or TNFRSF9/TNFSF9) are among the most explored and exploited TNFSF costimulatory pathways, mainly because of their central role in the initiation (CD40) and activation (4-1BB) of adaptive immune responses.

Moreover, the anti-tumoral activities of agonist molecules of CD40/4-1BB pathways have been evidenced in numerous preclinical models, but their clinical application has been hampered by tolerability issues mainly due to the engagement of the target outside the tumor (on-target off-tumor effects). This restricted therapeutic window makes these agonist molecules the payloads of choice for vectorization into oncolytic viruses that replicate specifically into tumors and, therefore, deliver the therapeutic proteins at active concentrations intratumorally while maintaining low systemic concentrations (Semmrich et al., 2022).

The signaling through most of the tumor necrosis factor receptor superfamily (TNFRSF) is physiologically obtained by clustering of receptors at the cell membrane (Bremer, 2013; Kucka and Wajant, 2020). The CD40 ligand (CD40L) and 4-1BB ligand (4-1BBL) are type 2 membrane proteins that assemble into a homotrimer and interact in trans with their cognate receptors. Those trans-interactions form clusters of receptors at the cell surface, leading to signaling. Receptor clusterization and activation can be also obtained artificially using agonist antibodies or TNFSF–Fc fusion proteins. These molecules, currently being evaluated in clinical trials, interact with their receptors by their paratope (mAb), or the ligand domain, and induce their clusterization by trans-interaction with Fc receptors (FcR).

To prevent off-target interactions with widely distributed FcR-containing cells, the Fc domain of the TNFRSF agonist can be replaced with a protein more strongly associated with tumor cells. Those molecules are ideally tumor-associated or -enriched cell surface antigens (TAA or TEA). In this case, the active molecule is then a fusion protein consisting of a TNFRSF agonist domain (antibody or ligand) fused to a TAA/TEA-binding moiety.

For instance, Pandey et al. (2021) and Medler et al., (2022) successfully constructed potent CD40 agonists by fusing the CD40L ectodomain or anti-CD40 single-chain fragment variable (scFv) from the agonist mAb to the anti-PDL1 mAb or scFv. Likewise, 4-1BB engagement was obtained by equivalent means (Fellermeier et al., 2016) or by fusing an antagonist anti-PDL1 single-domain antibody (sdAb) to agonist anti-4-1BB sdAb (Zhai et al., 2021). Many TAAs or TEAs have been used to dock agonist the TNFRSF on tumor cells or cells present in the tumor microenvironment (TME); however, PDL1 is particularly interesting as its expression increases in tumor treated by oncolytic virus (Liu et al., 2017).

Oligomerized ligands can also be used to activate the TFNR receptor independent of the cellular environment. Indeed, the oligomerized ligand alone is sufficient to induce clusterization and the subsequent activation of the TNFRSF (Wyzgol et al., 2009).

The examples of oligomerization of the TNFSF are far less abundant than those of Fc or anti-TAA/TEA fusions. Haswell et al. (2001) and Stone et al. (2006) reported a 12-mer (dodecamers) CD40 agonist by fusing surfactant protein D (SP-D) oligomeric domains to the CD40L ectodomain. This chimeric molecule, when fused to an antigen, increased its immunogenicity *in vivo* (Stone et al., 2006).

**FIGURE 1:**
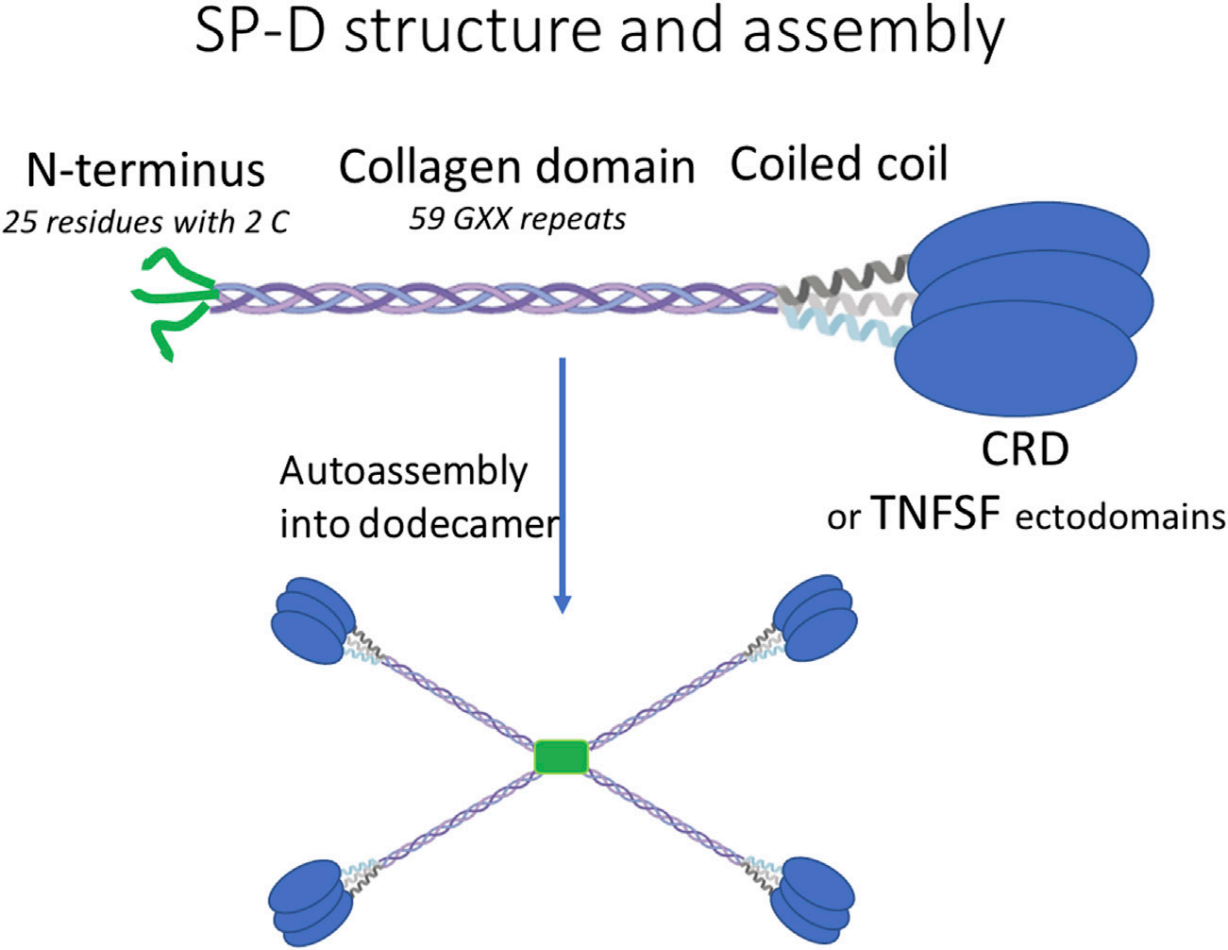
Descriptions of the general structure and auto-assembly of human SP-D and SP-D–TNFSF fusions.

The SP-D molecule contains three domains (N terminus peptide, collagen domain, and coiled coil) that together participate in successive oligomerization steps (Figure 1). Collagen and coiled coil trimerize, and the trimers further auto-assemble into mainly the dodecamer (i.e., tetramers of trimers) via the N-terminus peptide (i.e., the 25 first residues of mature SP-D, Figure 1). This N-terminus peptide contains two cysteines that ultimately lock the dodecamer by inter-molecular disulfide bonds. The SP-D dodecamer has a cross shape (Arroyo et al., 2018) in which the length of the branch is proportional to the number of GXX repeats of the collagen domain (White et al., 2008). While the collagen domain determines the size of the entire molecule, it is not essential for its oligomeric assembly. Indeed, the deletion of the entire collagen domain decreased the size of SP-D from 100 to 10 nm without affecting its dodecameric structure (White et al., 2008). The globular carbohydrate recognition domain (CRD) at the C-terminus of SP-D does not play any role in this oligomerization process and can be replaced by a protein, especially if this one is an obligate homotrimer like all members of the TNFSF (Figure 1), (Kanagavelu et al., 2012).

We report here the selection and vectorization into the vaccinia virus of an anti-PDL1 sdAb with an optimal PDL1 blocking format. We also report the generation of CD40 or 4-1BB agonist molecules by the fusion of their ligand ectodomains to either the anti-PDL1 sdAb or SP-D oligomerization domains. In the case of anti-PDL1 TNFSF fusions, the TNFRSF agonist activity was dependent on the presence of PDL1-expressing cells. Moreover, the optimal SP-D TNFSF format was a molecule with a shortened collagen domain comprising between 0 and 12 GXX repeats out of the 59 originally reported (Stone et al., 2006).

## Materials and methods

### Cells and viruses

HeLa (ATCC; CCL-2) is a human cervix tumor cell line. Cells were maintained in Dulbecco’s modified Eagle medium (DMEM, Gibco ref. 41966-029); glutamine 2 mM; gentamicin 40 μg/mL; and 10% fetal bovine serum (FBS).

Hs746T (ATCC; HTB-135) is a hypertriploid human lung adenocarcinoma cell line, epithelial in morphology and adherent. Cells were maintained in DMEM supplemented with 20% FBS and gentamycin 40 μg/mL.

HT29 (ATCC; HTB-38) is a human colorectal adenocarcinoma cell line. Cells were maintained in McCoy’s 5a medium (ATCC; 302007) supplemented with 10% FBS and gentamycin 40 μg/mL.

A549 (ATCC; CCL-185) is a lung carcinoma cell line. MIA PaCa-2 (ATCC; CCL-1420) is a human pancreatic cancer cell line. Those cells were maintained in DMEM supplemented with 10% FBS and gentamycin 40 μg/mL.

All viruses in this report were the vaccinia virus Copenhagen strain deleted of the thymidine kinase gene (*J2R*) and the ribonucleotide reductase gene (*I4L)* (Foloppe et al., 2019). For details on the generation of recombinant viruses, see “sdAb vectorization” and “SP-D and anti-PDL1 TNFSF fusions.” Recombinant viruses were obtained by homologous recombination at the loci J2R and I4L of a parental virus in which viral genes were initially replaced by markers coding fluorescent proteins. The selection of recombinant viruses was achieved by picking non-fluorescent lysis plaques. The I4L locus was only used when two transgenes were inserted, e.g., light and heavy chains of avelumab. Transgene transcription was under the control of the early/late pH5R poxvirus promoter. Viruses were produced and purified as described previously by Semmrich et al. (2022).

### Alpaca immunizations, isolation, and humanization of anti-human PD-L1 blocking sdAb

Six male alpacas were immunized with recombinant human PDL1 (Randox). All animals were immunized at monthly intervals for 7 months. Lymph node cells were harvested, and single-domain antibody libraries were generated by RT–PCR using published primer sets (Harmsen et al., 2000; Maass et al., 2007). DNA libraries were ligated into the pScreen expression vector and transformed in Top10 *E. coli* competent cells (Invitrogen) for expression. Antibody clones binding PD-L1 were subsequently isolated by ELISA screening against immobilized human recombinant PD-L1 (Randox and R&D Systems).

In total, 81 sdAb clones were isolated and sequenced and 24 were subsequently selected on the basis of having substantially different DNA sequences to undergo scaled-up expression in *E. coli* and characterization assessment. In brief, colonies of each clone were inoculated into 200 mL of TB media (Melford) and grown up at 37 °C. The following day, cultures were lysed using BugBuster cell lysis reagent and purified by immobilized metal affinity chromatography (IMAC) using TALON resin (TaKaRa). SdAb preparations were then quantified at 280 nm, and 2 µg of each antibody was electrophoresed on a 12% polyacrylamide gel via standard methods. A PD1/PDL1 inhibition assay was developed to test all 24 clones for inhibition of the PD1/PDL1 interaction. In short, an ELISA plate was coated overnight at 4°C with 1 μg/mL of recombinant human PD1 (Randox) in PBS pH 7.4. The following day, after a blocking step, 50 µL of 60 ng/mL of PDL1-Fc (R&D Systems, 156-B7) was added to each well and incubated for 1 h at 37 °C. Following incubation, 50 µL per well of serially diluted (10,000–0.1 ng/mL) anti-PDL1 sdAb clones were added to test the blocking of the PD1/PDL1 interaction. The plate was incubated at 37°C for 1 h. This was followed by the addition of 1/1,000 dilution of goat anti-human IgG horseradish peroxidase (HRP) conjugate (Abcam Ab97225) detector antibody. The signal was then developed by adding 50 µL/well of 3,3′,5,5′tetramethylbenzidine (TMB) reagent and incubating at room temperature for 20 min in the dark. Signal development was stopped by the addition of 50 µL/well of 2N sulfuric acid. The optical density of each well was read at 450 nm.

The lead anti-PDL1 clone 32.1A1 with the following complementarity determining regions (CDR): CDR1: RTFREYGMG; CDR2: TISSSGSYSY; and CDR3: AASSLLRGSSSRAESYDS gave the best blocking effect. 32.1A1 was humanized by grafting its CDRs into a model human VH3 framework sequence, and the humanized sequence was synthesized and cloned into the expression vector pScreen for production and characterizations as described previously. The resulting humanized sdAb was named GS542.

### sdAb vectorization

All protein constructs were inserted in the vaccinia virus transfer plasmid under the control of the early/late poxvirus promoter pH5R. All constructs were fused to the heterologous signal peptide MGWSCIILFLVATATGVHS and contained a 6xHis tag at their C-terminus. Those constructs were inserted in the vaccinia virus genome at the TK locus (except avelumab for which TK and RR loci were used; see the following section for details).

“VV–monomeric” construct encodes GS542 (also named sdAb in figures and text) alone.

“VV–single-chain dimeric” encodes two fused GS542 separated by a (GGGGS)_3_ linker.

“VV–dimeric hinge” encodes GS542 fused to the hinge of human IgG2 (ERKCCVECPPCP) that dimerizes via intermolecular disulfide bonds.

“VV–Fc fusion” encodes GS542 fused to the Fc of human IgG1 (accession P01857) with the cysteine that forms the inter disulfide bond with the light chain changed into serine (EPKSSDKTHTC….).

“VV–avelumab” encodes light and heavy chains of avelumab, respectively, at RR and TK loci, respectively. The heavy chain was fused to 6xHis tag at its C-terminus.

### SP-D and anti-PDL1 TNFSF fusions

All protein constructs were inserted in the vaccinia virus transfer plasmid under the control of the early/late poxvirus promoter pH5R. All constructs were fused to the heterologous signal peptide MGWSCIILFLVATATGVHS and contained a FLAG tag at their C-terminus (except “CD40L”). Those constructs were inserted in the vaccinia virus genome at the TK locus.

“CD40L” construct encodes the CD40L (G116 to L261 from P29965) ectodomain.

“4-1BBL” construct encodes the 4-1BBL (D80 to E254 from P41273) ectodomain.

“sdAb–CD40L” construct encodes humanized anti-human PD-L1 sdAb (GS542) fused to the CD40L (G116 to L261) ectodomain.

“sdAb–link–CD40L” is like “sdAb–CD40L” but has a (GGGS)_3_ linker between GS542 and CD40L.

“sdAb–link–4-1BBL” is like “sdAb–link–CD40L” but the with 4-1BBL ectodomain (D80-E254) replacing CD40L.

Human SP-D (accession number: P35247) with different truncations of the collagen domain were fused at the N-terminus of human CD40L (G116 to L261) or 4-1BBL (D80 to E254) extracellular domains as follows:

“Full-length” construct encodes human SP-D oligomeric domains A21 to G257, including the N-terminus peptide, collagen domain (i.e., 59 collagen GXX repeats), and coiled coil fused to CD40L (N119 to L261). For clarity, this construct corresponds to SP-D molecules with the CRD replaced by the CD40L ectodomain.

“0R Col” encodes human SP-D oligomeric domains entirely deleted of the collagen domain (0 repeat) (i.e., A21 to S45, D223 to E252) fused to CD40L as described previously.

“0R Col linker” is like “OR Col” but a (GGGS)_3_ linker was inserted between SP-D and CD40L.

“12R Col” encodes human SP-D with a shorten collagen domain containing 12 consecutive GXX repeats (G79 to P114) selected using the online collagen stability calculator https://compbio.cs.princeton.edu/csc/peptide.cgi (SP-D part is then composed of A21 to S45, G79 to P114, and D223 to E252) fused to CD40L as described previously.

“12R Col linker” is like “12R Col,” but a (GGGS)_3_ linker was inserted between SP-D and CD40L.

The other CD40L constructions are described in Table 1 and are like “12R Col” except for their collagen domain length and sequence (see Table 1 for details).

**TABLE 1:** List of SPD-CD40L fusions generated for this publication.

Construct	N-terminus*	Collagen	Coiled coil*	GS linker
Full-length SPD	Yes **	59 repeats (46–222)	Yes **	No
12R Col	Yes	12 repeats (79–114)	Yes	No
12R Col linker	Yes	12 repeats (79–114)	Yes	(GGGS)x3
0R Col	Yes	No	Yes	No
0R Col linker	Yes	No	Yes	(GGGS)x3
6R Col NGly	Yes	12 repeats (79–96)	Yes	No
6R Col	Yes	12 repeats (97–114)	Yes	No
12bisR Col	Yes	12 repeats (121–156)	Yes	No
19R Col	Yes	19 repeats (79–135)	Yes	No
30R Col	Yes	30 repeats (46–135)	Yes	No

*All constructs contained the same human SPD N-terminus and coiled coil and CD40L ectodomain at their C-terminus.

**Numbering according to human SPD (P35247): N-terminus (peptide 22–45) and coiled coil (peptide 223–252).

“12R Col 4-1BBL” is like “12R Col” but with the 4-1BBL ectodomain (D80-E254) replacing CD40L.

### Infection/transfection experiments

Infection/transfection of HeLa cells was carried out with the goal of selecting the most effective TNFSF constructions to be vectorized in the VV genome.

In brief, cells were seeded 2 days prior to infection at 4E+05 cells/well/3 mL of complete medium (DMEM Gibco, 41966-029; glutamine 2 mM; gentamicin 40 μg/mL; 10% FBS) in six-well plates.

Cells were infected at MOI 1 with a double-deleted vaccinia virus Copenhagen strain encoding no transgene. After 30 min at room temperature (RT), the viral inoculum was removed and replaced by 1.2 mL of complete medium without FBS. The plates were incubated for 2 h at 37 °C with 5% CO_2_. Transfection was then performed by the addition of 1 µg of plasmid DNA formulated with 4.5 µL of Lipofectamine 2000 (Invitrogen, 11668-027) in each well, following the manufacturer’s protocols. Plasmids encoding irrelevant proteins (either murine T-cell engager with a FLAG tag or green fluorescent protein) were used as negative controls. The infection/transfections were performed in triplicate. The plates were incubated 48 h at 37 °C and 5% CO_2_. The culture supernatants were then collected, centrifuged, and filtrated on 0.1 µm filters to remove all virus particles and most of cellular debris. The clarified supernatants were stored at −80 °C until use.

### Vectorization and virus production

Recombinant viruses were generated using the same plasmids used for the infection/transfection experiment described previously. In brief, chicken embryo fibroblasts (CEFs) were infected with parental viruses encoding GFP at the J2R (TK) locus and deleted from the I4L (RR) gene or encoding mCherry at the RR locus (the latter for avelumab vectorization only). Infected cells were transfected with the transfer plasmid carrying the expression cassette flanked by recombination arms (DNA sequence homologs of upstream and downstream TK or RR loci). Recombinant viruses were selected using the binocular microscope by picking “white” (i.e., mCherry and/or GFP negative) lysis plaques. The expression cassette was verified by PCR amplification followed by DNA sequencing.

Recombinant viruses were amplified in CEF and kept at +4 °C until use.

### Expression experiments using recombinant viruses

HeLa or A549 (for experiments with TNFSF fusions) or A549, HT29, and MIA PaCa2 (for the sdAb vectorization experiment) cells were seeded in six-well plates at 1.5E+06 cells/wells/2 mL of complete medium the day prior to infection. Cells were infected at MOI 0.1 with one of the indicated viruses. After 30 min of incubation, the culture medium was discarded and replaced by 2 mL of DMEM; glutamine 2 mM; and gentamicin 40 μg/mL. Cells were incubated for 48 h at 37 °C with 5% CO_2_, and then, the culture supernatants were recovered and treated as described previously.

### Immunoblots

25 µL of samples (clarified supernatants) from infections/transfections or infections were treated using Laemmli buffer (Bio-Rad, 161-0747) with (reducing) or without (non-reducing) beta-mercaptoethanol. In the case of reducing conditions, samples were heated at 95 °C for 3 min. Samples were then loaded on polyacrylamide gel (TGX 4%–15% Stain-Free Bio-Rad), and migration was performed in Tris-glycine-SDS buffer (Bio-Rad, 161-0772). Western blot was performed using the Trans-Blot Turbo System (Bio-Rad), set up on midi program high molecular weight. Blots were then incubated with either anti-His-HRP (QIAGEN, 34460) or anti-FLAG-HRP-conjugated antibody (Sigma A8592) or rabbit anti-CD40L antibody (LSBio, LS-C293374) at 2 μg/mL followed by goat anti-rabbit HRP (Dako P0448) at 0.15 μg/mL in the case of CD40L detection. The iBind Flex Western System (Invitrogen, SLF 2000) was used for all antibody incubations. Blots were incubated using HRP substrate (Amersham ECL Prime Western blotting detection), and luminescence was recorded by using ChemiDoc apparatus (Bio-Rad).

### PD1/PDL1 competition ELISA for culture supernatants from transfections and/or infections

Human PD1 Fc (R&D systems, 1086-PD) was coated on the 96-well ELISA plate at 0.25 μg/mL in 50 mM carbonate buffer at pH 9.6. Solutions or culture supernatants containing the PDL1 blockers were added and then two-fold serially diluted on the plate. Human biotinylated PDL1 (R&D systems, BT-156) at 0.2 µg/mL was added to all wells. PD1/PDL1 complex formation was detected by the addition of HRP-conjugated streptavidin (SouthernBiotech, 7105-05) diluted 5000-fold. Finally, HRP substrate TMB was added to each well, and the enzymatic reaction stopped after 20 min by the addition of 2M sulfuric acid. Absorbance at 450 nm was measured using the TECAN microplate reader, and optical density (OD) at 450 nm *versus* dilution (e.g., 0.1 meaning supernatant diluted 10-fold) of culture supernatants was plotted using GraphPad Prism software.

### PD1/PD-L1 blocking bioassay

PD-1/PD-L1 blockade bioassay (Promega, J1250) was used to measure the potency of antibodies to block the cellular PD1/PDL1 interaction and following manufacturer’s instructions. Fold induction of luciferase activity was measured by luminescence using Berthold reader and MikroWin 2000 software following: fold change = (RLU sample – RLU background)/(RLU control- RLU background). RLU = relative luminescence unit. Fold induction *versus* concentration of antibody or dilution of supernatant were plotted using GraphPad prism. The curves were fitted with the five-parameter logistic equation, and the inhibitory concentration (IC50) or inhibitory dilution (ID50) values were determined.

CD40-binding ELISA method CD40 Fc was coated on the MediSorp (Nunc) 96-well ELISA plate at 0.5 μg/mL in 50 mM carbonate buffer at pH 9.6. Clarified supernatants of the infection/transfection experiments were diluted 10-fold, added to the first well of the ELISA plate, and further diluted two-fold serially in ELISA saturation buffer directly on the plate. The bound CD40L was detected by adding a non-competitive anti-human CD40L monoclonal antibody (Bio-Rad, MCA1561) at 1 μg/mL in saturation buffer. Anti-mouse immunoglobulin HRP-conjugated antibody (Dako, P0447) at 0.5 μg/mL was then added to each well. Finally, development was performed as described previously. OD 450 nm *versus* dilution of culture supernatants was plotted using GraphPad Prism software. Curves were fitted using the five-parameter logistic equation.

### CD40 agonist bioassay

HEK-Blue CD40L cells (InvivoGen, hkb-cd40) are recombinant cells transformed to express both the human CD40 and a reporter enzyme (secreted embryonic alkaline phosphatase: SEAP) under the transcriptional control of a NF-κB-inducible inducible promoter. Upon activation of CD40, SEAP is produced, and its enzymatic activity was measured in the culture medium following the manufacturer’s recommendations. In brief, 50,000 HEK-Blue CD40L cells with or without 50,000 PDL1-positive human tumor cells (Hs746T) were distributed in 96-well plates and incubated with 20 µL of serial dilutions of clarified supernatants generated by the infections or infection/transfections described previously. After 24 h of incubation at 37 °C and 5% CO_2_, 40 µL of the culture medium was transferred using 160 µL of SEAP substrate (InvivoGen, hb-det2) and incubated for 3 h at 37 °C. Absorbance at 620 nm was measured using a microplate reader, optical density *versus* 1/supernatant dilution was plotted using GraphPad Prism software, and four-parameter logistic curve analysis was performed.

In one experiment, avelumab, or an irrelevant human IgG1, was added at 100 μg/mL to the mix of HEK-Blue CD40L and Hs746T cells to demonstrate the specificity of PDL1 involvement in the CD40 agonist activity of the sdAb–link–CD40L fusion.

### 4-1BB agonist activity

The 4-1BB Bioassay Promega kit (JA2351) was used according to the manufacturer’s instructions. In brief, 25 µL of effector cells/well were mixed with 25 µL medium or PDL1-positive (Hs746T) cells (50,000 cells/25 µL). Then, 25 µL of serial dilutions of clarified supernatants were added to each well. Cells were then incubated at 37°C at 5% CO_2_ for 6 h. 75 µL/well of reconstituted Bio-Glo (Promega, G7941) was added, and luminescence was recorded using Berthold reader and MikroWin 2000 software. Luminescence *versus* 1/supernatant dilution was plotted using GraphPad Prism software, and four-parameter logistic curve analysis was performed.

### Statistical analysis

Analysis was performed using SAS 9.4. EC50 was log-transformed to fulfill Gaussian distribution. A mixed model with cell type as random effect was used to compare the five viruses presented in Supplementary Figure S3.

## Results

### Generation, vectorization, and selection of the best anti-PDL1 sdAb format

**FIGURE 2:**
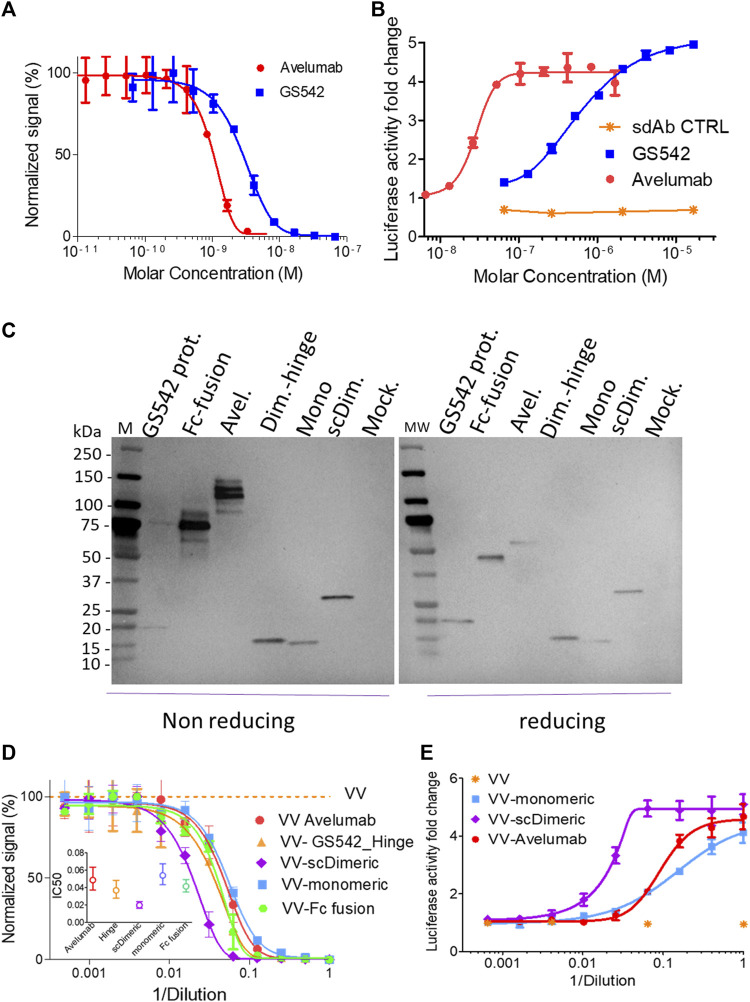
Single-chain dimeric anti-hPDL1 sdAb GS542 is the best vaccinia virus vectorized constructs for blocking PD1/PDL1 interactions. Purified sdAb (also mentioned as GS542) or avelumab **(A, B)** or the culture medium of A549 or MIA PaCa2 cells infected by the indicated recombinant virus (i.e., VV-xx: **(C–E)** were analyzed for transgene expression by immunoblot using an HRP-conjugated anti-6xHis tag monoclonal antibody and **(C)** competition of PD1/PDL1 interaction either by ELISA **(A, D)** or using cellular bioassay **(B, E)**. Inserted graph in Figure 2D represents the calculated effective concentration 50% (EC50) +/- confidence interval (CI) 95% for each curve. Mock: culture medium of cells infected with a VV encoding no transgene; scDim: VV encoding single-chain dimeric sdAb GS542; Mono: VV encoding monomeric sdAb GS542; Avel: VV encoding avelumab; Dim. Hinge: VV encoding GS542 fused to IgG2 hinge; and Fc fusion: VV encoding sdAb fused to human IgG1 Fc.

sdAbs recognizing human PDL1 were generated by immunization of alpacas with PDL1 recombinant protein at a licensed Randox facility. An sdAb phage library was constructed from lymphocyte RNA and panned on immobilized PDL1 (Randox). Selected sdAbs were produced in *E. coli* and then assessed for their ability to bind PDL1 (Supplementary Figure S1) and disrupt the PD1/PDL1 interaction. The best blocker was humanized (GS542), and its ability to block the PD1/PDL1 interaction was verified and remained intact both in ELISA and cellular assays (Figures 2A,B).

Several formats of GS542 (also named sdAb) were then vectorized in vaccinia virus to obtain the best PD1/PDL1 blocking activity in the culture supernatant of infected tumor cells. GS542 was vectorized either alone (monomeric), fused to itself (single chain dimeric), fused to the human IgG1 Fc domain (Fc fusion), or fused to IgG2 hinge (dimeric-hinge). The three latter constructs aimed to generate bivalent constructs to increase its apparent avidity for PDL1. All the recombinant viruses replicated as efficiently as the benchmark virus (virus without any transgene, Supplementary Figures S2A, B).

Three different human tumor cells (A549, HT29, and MIA PaCa2) were infected by different recombinant viruses, and their culture supernatants were analyzed for transgene expression using anti-His tag mAb. All constructs were well expressed (Figure 2C), and those that form interchain disulfide bonds migrated with the expected pattern under the non-reducing condition, except for the sdAb–hinge construct, which appeared mainly as a monomer instead of the expected dimer (Figure 2C). The PD1/PDL1 blocking activities of different culture supernatants were then tested by ELISA and compared. Although, avelumab has a better blocking activity than sdAb as purified protein (in mole at least, Figures 2A,B), the blocking activity of culture supernatants of cells infected by VV–monomeric and VV–avelumab were quite similar indicating a probable higher expression of sdAb (Figure 2D). Interestingly, VV–single-chain dimeric generated the culture medium with the best blocking activity and for the three tumor cell lines tested (Figure 2D, Supplementary Figure S3). The two other constructs, dimeric hinge and Fc fusion, despite a clearly dimeric format for the latter, did not bring any improvement in the PD1/PDL1 blocking activity compared to sdAb monomeric (Figure 2D, Supplementary Figure S3). Therefore, the evaluation of those two viruses was discontinued.

The same supernatants were then assessed for their PD1/PDL1 blocking capacities using transformed cells expressing PD1 or PDL1 at their surface. The results obtained with this biological assay, and for samples of the three tumor cell lines tested, confirmed those obtained in competition ELISA: vectorization of single-chain dimeric sdAb (scDimeric) gave the best PD1/PDL1 blocking activity among all PDL1 blockers assessed (Figure 2E) and without impairing virus replicative or oncolytic properties (Supplementary Figure S2A, C).

### Generation of sdAb-TNFSF fusions with PDL1 dependent agonist activity

**FIGURE 3:**
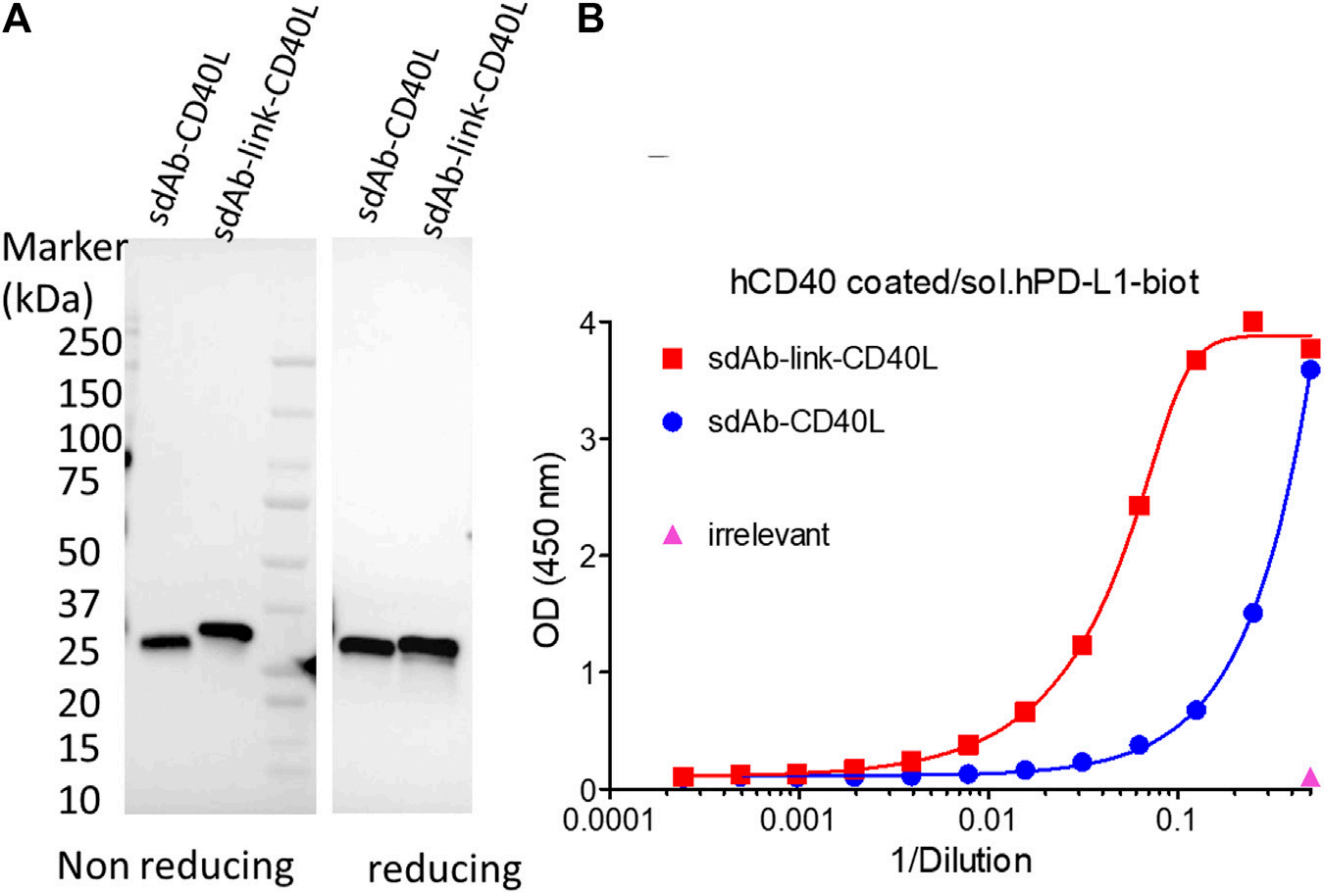
GS542–CD40L fusions are expressed and bind both CD40 and PDL1. Culture medium of HeLa cells infected/transfected by plasmids encoding the indicated GS542–CD40L fusions (also named sdAb–CD40L) were analyzed for transgene expression by immunoblot using an HRP-conjugated anti-FLAG tag monoclonal antibody **(A)**; the ability of the expressed fusion to bind simultaneously CD40 and PDL1 proteins by ELISA **(B)**. SdAb–CD40L and sdAb–link–CD40L refer to the fusion of GS542 sdAb directly to the CD40L ectodomain or through a (GGGS)_3_ linker, respectively.

**FIGURE 4:**
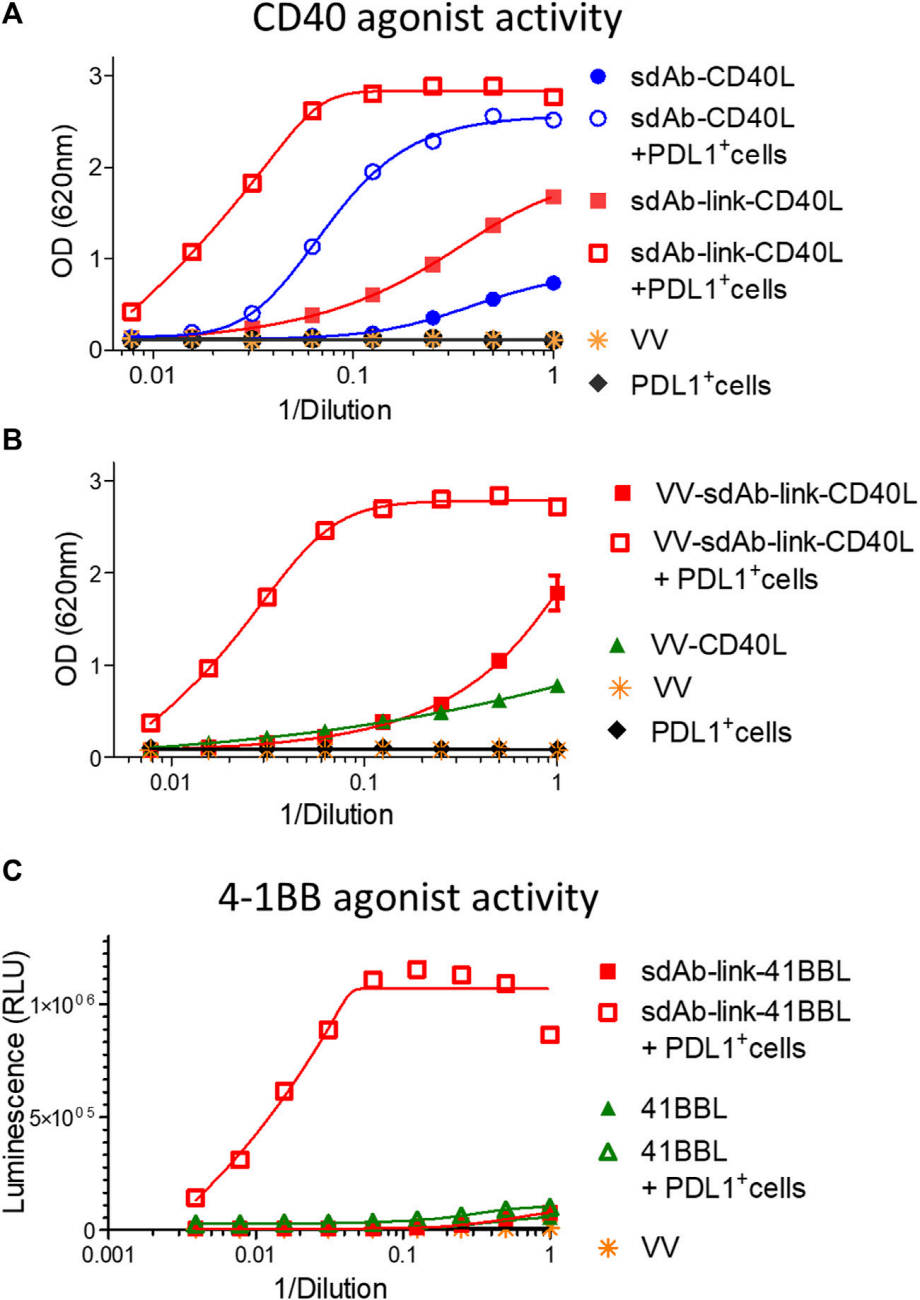
GS542–linker–TNFSF fusions are potent TNFRSF agonists in the presence of PDL1^+^ cells only. Culture medium of HeLa cells either infected by the indicated recombinant virus **(B)** or infected/transfected by indicated plasmids **(A, C)** encoding different GS542–TNFSF fusions were analyzed for their CD40 agonist activity using InvivoGen HEK-blue CD40L cells **(A, B)** or for their 4-1BB agonist activity using Promega 4-1BB effector cells **(C)**. SdAb–CD40L and sdAb–link–CD40L refer to the fusion of GS542 sdAb directly to the CD40L ectodomain or through a (GGGS)_3_ linker, respectively. Accordingly, VV–sdAb–link–CD40L is a vaccinia virus encoding the sdAb–link–CD40L construct. SdAb–link–4-1BBL refers to the fusion of GS542 sdAb to the 4-1BBL ectodomain through a (GGGS)_3_ linker.

GS542 was then fused to the N-terminus ectodomain of the TNFSF member such as CD40L and 4-1BBL. Two versions of the bispecific molecule were designed for sdAb–CD40L by the presence or absence of a (GGGS)_3_ linker between the two moieties. The expression and CD40 agonist activity of these different constructions were assessed on culture supernatants of infected/transfected cells. HeLa cells were infected with empty vaccinia virus and transfected with the transfer plasmids carrying different sdAb–CD40L constructions under the control of the same early/late poxvirus promoter (i.e., pH5R). As shown in Figure 3A, the two formats were equally expressed and were able to bind PDL1 and CD40 simultaneously on a sandwich ELISA (Figure 3B). However, the version with a linker had a better binding curve with a ∼six-fold decrease in EC50 compared to the version without the linker. Those samples were then tested on HEK-Blue CD40L cells that express a reporter phosphatase under the control of a CD40 inducible promoter and in the presence or absence of PDL1-expressing tumor cells (i.e., Hs746T). Figure 4A shows that both fusion polypeptides had weak CD40 agonist activity on their own. However, their CD40 agonist activities were greatly enhanced in the presence of PDL1-expressing cells demonstrating the conditional (i.e., the presence of PDL1) activation of CD40 of those molecules. The sdAb–linker–CD40L construct with the best CD40 agonist activity was then vectorized by insertion at the TK locus of vaccinia virus. This virus had the same replication and oncolytic activity as benchmark virus (Supplementary Figures S2A, C) indicating that the transgene did not significantly interfere with the viral cycle. Moreover, the results obtained after infections with recombinant virus were like those of infection/transfection experiments (Figure 4B) confirming that the vectorization of sdAb–linker–CD40L allows the production of a potent PD-L1-dependent CD40 agonist in the environment of infected tumor cells. Furthermore, to extend the principle to another member of the TNFSF, CD40L was exchanged for a 4-1BBL ectodomain to generate an sdAb–linker–4-1BBL construct that was tested directly after infection/transfection in a 4-1BB bioassay. In those conditions, 4-1BBL or sdAb–linker–4-1BBL were expressed at the same level (Supplementary Figure S4) and did not induce significant 4-1BB agonist activity by themselves as expected. However, in the presence of PDL1-expressing tumor cells, the 4-1BB agonist activity was dramatically increased for sdAb–linker–4-1BBL only (Figure 4C).

**FIGURE 5:**
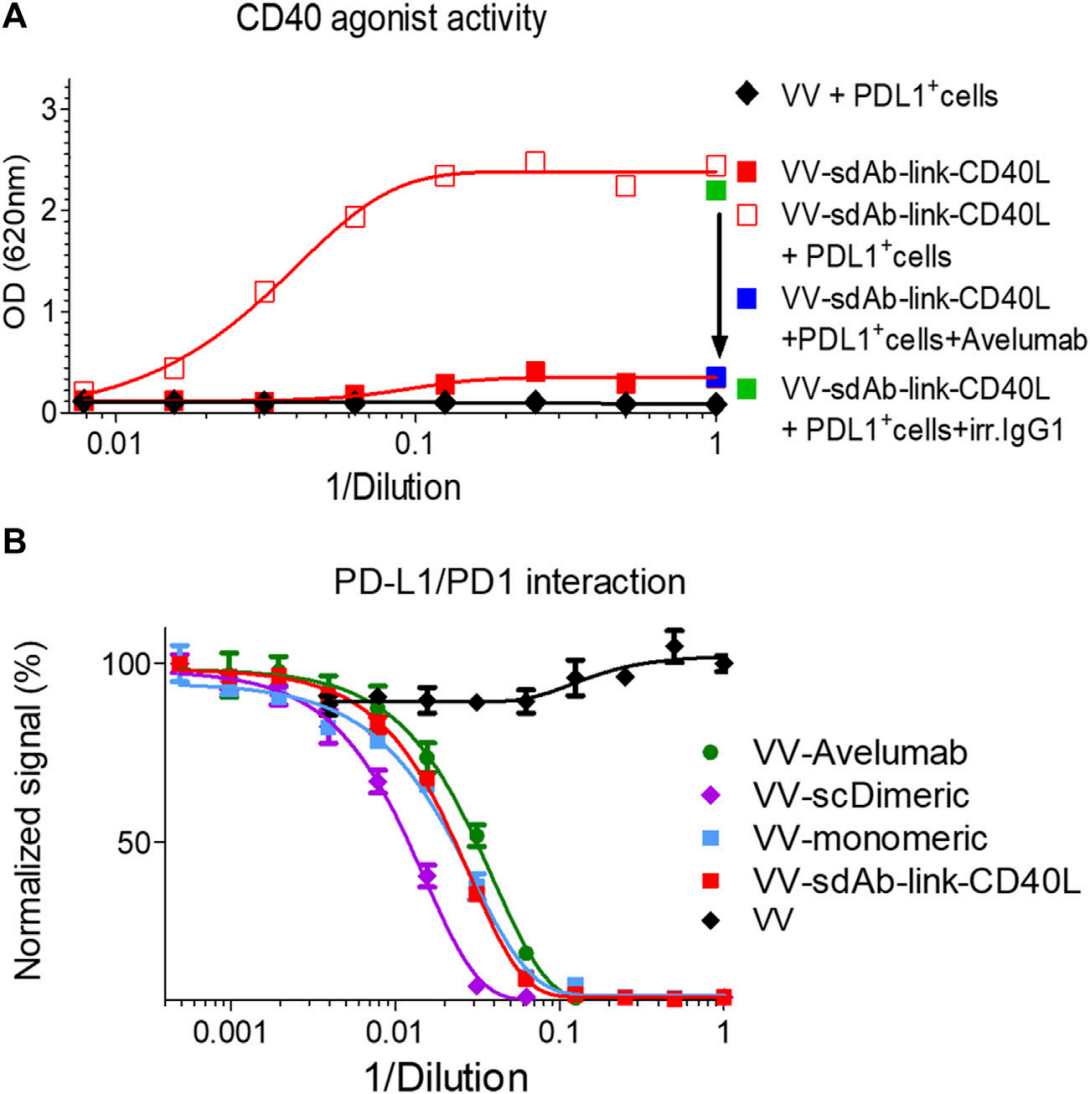
The CD40 agonist activity of sdAb–linker–CD40L is inhibited by coincubation with a PDL1 blocking antibody, and sdAb–linker–CD40L has a PD1/PDL1 blocking activity. CD40 agonist activity of culture supernatants of VV–sdAb–linker–CD40L-infected cells was analyzed, as shown in Figure 4, in the presence or absence of 100 μg/mL of PDL1 blocking antibody (avelumab). In the case of the addition of avelumab, or of its isotype control, only undiluted supernatants were tested **(A)**. The PD1/PDL1 blocking activity of the culture medium of A549 cells infected by the mentioned virus was measured, as shown in Figure 2D **(B)**. VV–sdAb–link–CD40L is a vaccinia virus encoding the sdAb–link–CD40L construct. VV–scDimeric: VV encoding single-chain dimeric sdAb GS542; VV–monomeric: VV encoding monomeric sdAb GS542; and VV–avelumab: VV encoding avelumab.

To demonstrate the essential role of cell surface PDL1 in the activation of the TNFRSF by sdAb–CD40L fusion, the CD40 agonist assay was performed in the presence of a high concentration of avelumab that binds PDL1 at the same epitope as the sdAb (Supplementary Figure S5). Figure 5A shows that avelumab completely abrogates the agonist activity of sdAb–CD40L fusion.

Those results demonstrate that GS542–linker–TNFSF fusions are effective agonists of the TNFRSF only in the presence of PD-L1-positive cells.

Finally, the PD1/PDL1 blocking activity of sdAb once fused to CD40L was verified with the culture supernatant of A549 cells infected with vaccinia virus encoding GS542–linker–CD40L (VV–monomeric, VV–scDimeric, and VV–avelumab were added as benchmarks) and by using competition ELISA. Figure 5B shows that samples containing GS542–linker–CD40L or monomeric sdAb had roughly the same blocking activity, demonstrating that the fusion to CD40L did not impair GS542’s blocking activity. However, no gain of blocking activity was observed as might be expected for a putative trimeric sdAb–CD40L fusion.

### Construction and expression of the SP-D fusion proteins

An alternative to sdAb–TNFSF fusions to induce TNFRSF signaling is the direct use of the oligomeric soluble ligand. SP-D–TNFSF fusion fulfills this requirement (Haswell et al., 2001). To optimize both the level of expression and/or the CD40 agonist activity of SP-D–CD40L fusions, several constructs containing various sizes of collagen domains were generated (Table 1). The fragments of the collagen domain were selected based on their higher predicted stabilities of the collagen triple helix (Supplementary Figure S6). In the first generation of constructs, the length of the collagen arm was either untouched (59 GXX repeats: “full length”) or shortened to 12 and 0 repeats (“12R Col” and “0R Col,” respectively). For the latter two formats, two constructs with and without a (GGGS)_3_ linker between the coiled coil and the CD40L were generated.

**FIGURE 6:**
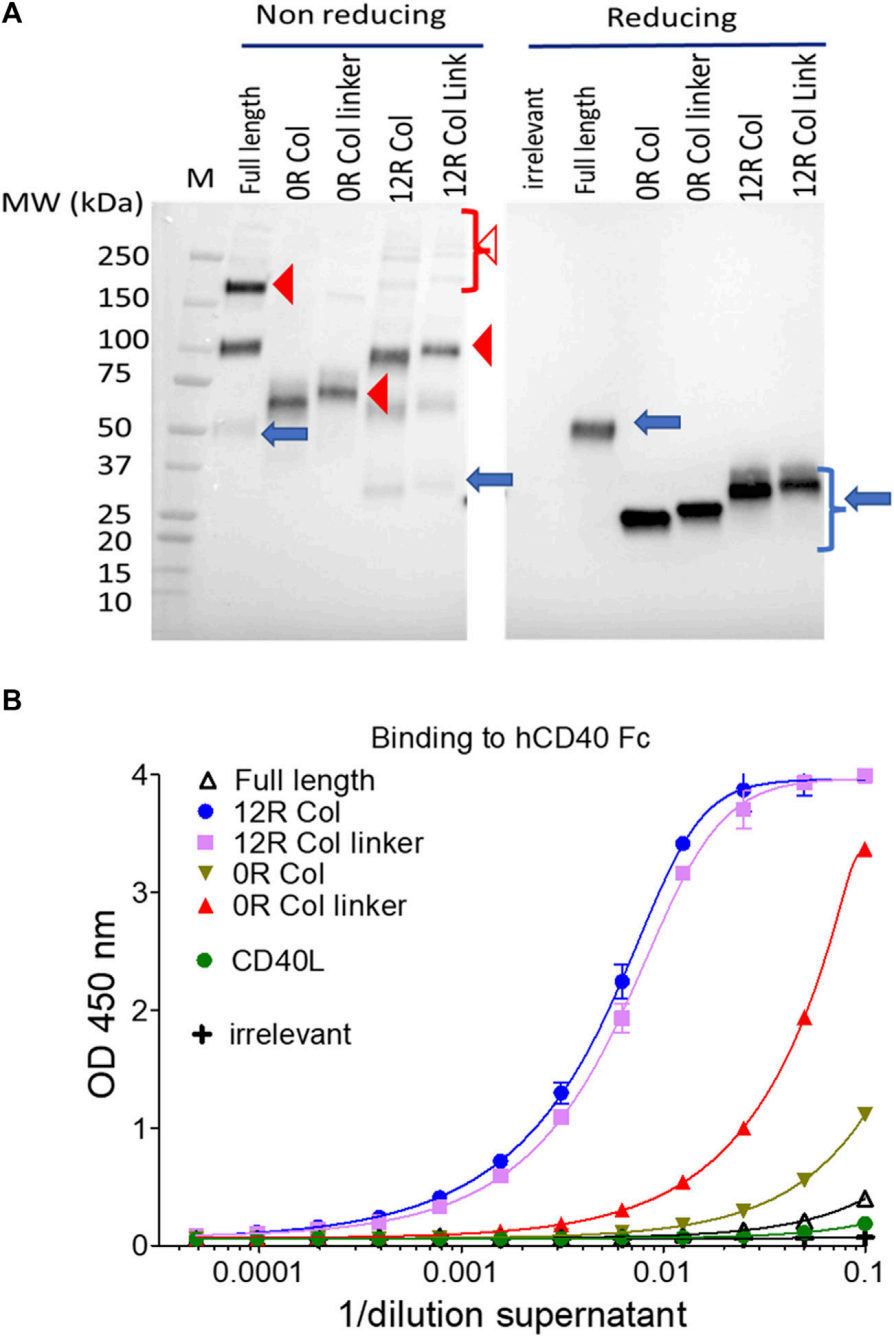
SP-D–CD40L fusions are all expressed at approximately the same level and bind CD40 *in vitro*. The culture medium of HeLa cells infected/transfected by plasmids encoding the indicated SP-D–CD40L fusions were analyzed for transgene expression by immunoblot, in both non-reducing and reducing conditions, and using an HRP-conjugated anti-FLAG tag monoclonal antibody **(A)**; the ability of the expressed fusions to bind immobilized CD40 was assessed by ELISA **(B)**. Blue arrows, closed, and open red arrowheads indicate monomers, trimers, and multimers, respectively **(A)**. Full length is the fusion of the entire human SP-D deleted of the CRD and fused to the human CD40L ectodomain. 12R COL is the fusion of oligomeric domains of human SPD containing only 12 collagen repeats and fused to the CD40L ectodomain. The 12R COL link is the same construct as previously described but with a (GGGS)_3_ linker between the C-terminus of the collagen domain and the N-terminus of the CD40L ectodomain. OR COL and OR COL link are the same constructions described previously except that they are lacking any collagen domain (zero collagen repeat). CD40L is a construction encoding the human CD40L ectodomain. For details on constructions, refer to Table 1 and Materials and methods.

The expression of these fusions into the culture supernatants of infected/transfected cells was evaluated first by immunoblot under reducing conditions (Figure 6A), demonstrating that all constructs were expressed at approximately the same level. Moreover, in non-reducing conditions, the construction without any collagen (“0R Col”) appeared almost exclusively as a trimer whereas the full length and 12 repeat constructs migrated as a mix of monomers to trimer forms with the presence of some high molecular weigth oligomers. The presence of the linker between the coiled coil and CD40L did not change the patterns of oligomerization (Figure 6A). Of note, the main limitation of immunoblots in these conditions was to capture only disulfide cross-linked multimers.

The ability of each construct to bind CD40 was then evaluated by ELISA using immobilized CD40 for capture and a non-competitive monoclonal anti-CD40L antibody for detection. In this assay, the binding of CD40L to CD40 was barely detectable (Figure 6B). The best binders were the SP-D fusions with 12 collagen repeats (12R Col) with or without linkers, followed by the construct without any collagen but with a linker (0R Col linker). Finally, the constructs without any collagen (0R Col) or with full-length collagen, which correspond to the two molecules with the extreme arm lengths, were relatively poor binders (Figure 6B).

In addition, those results indicate that all constructs were expressed as secreted molecules that assembled into trimers and higher forms of oligomers that were able to bind CD40 *in vitro*.

### Biological activity of SP-D–CD40L constructs

**FIGURE 7:**
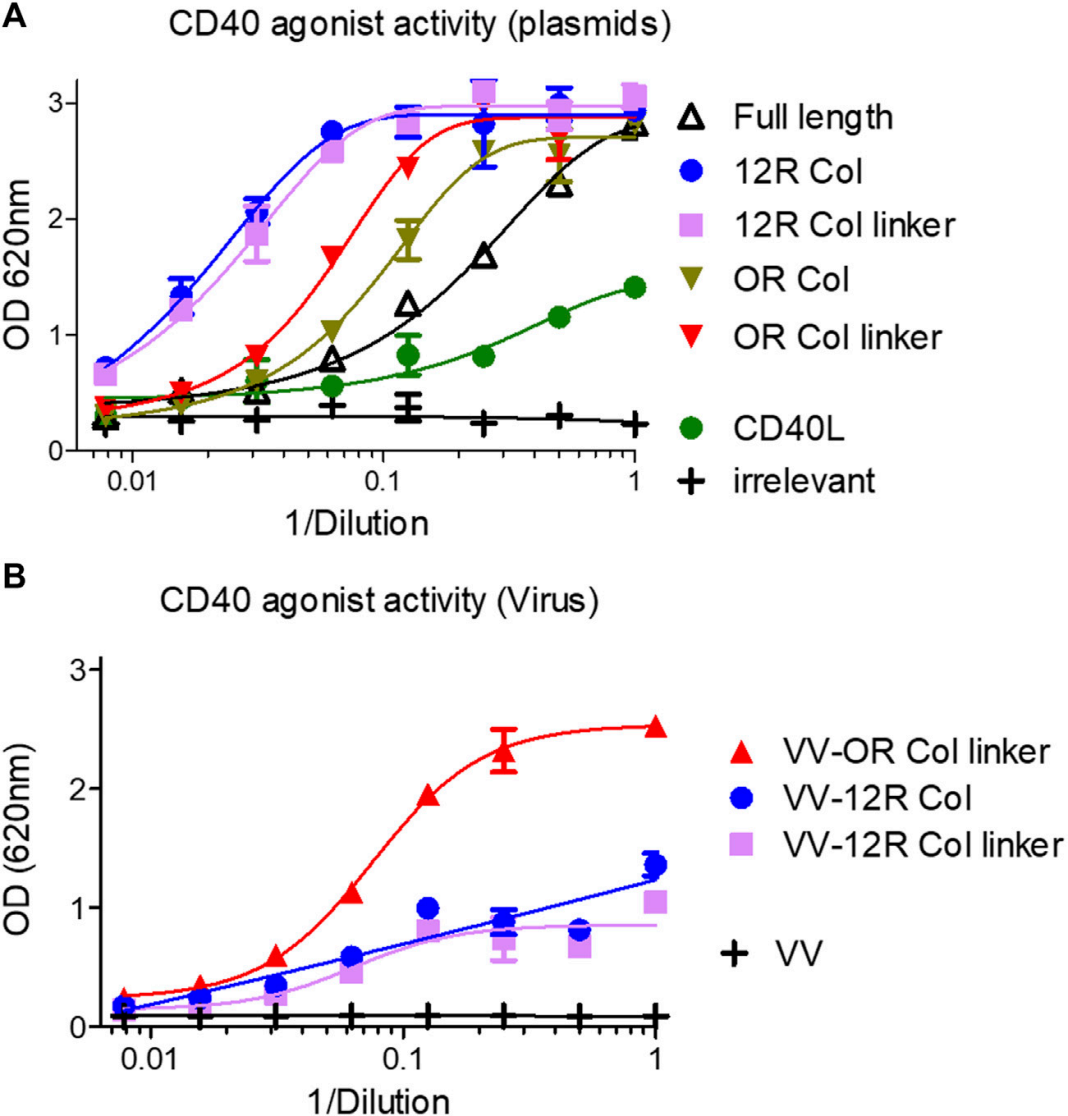
SP-D–CD40L fusions with shortened collagen domains are potent CD40 agonists. Culture medium of HeLa cells either infected by the indicated recombinant virus **(B)** or infected/transfected by indicated plasmids **(A)** encoding the different SP-D–CD40L fusions was analyzed for their CD40 agonist activity using InvivoGen HEK-blue CD40L cells **(A, B)**. Full length is the fusion of the entire human SP-D deleted of the CRD and fused to the human CD40L ectodomain. 12R COL is the fusion of oligomeric domains of human SPD containing only 12 collagen repeats and fused to the CD40L ectodomain. 12R COL link is the same construct as described previously but with a (GGGS)_3_ linker between the C-terminus of the collagen domain and the N-terminus of the CD40L ectodomain. OR COL and OR COL link are the same constructions described previously except that they are lacking any collagen domain (zero collagen Repeat). CD40L is a construction encoding the human CD40L ectodomain. For details on constructions, refer to Table 1 and Materials and methods.

The HEK-Blue CD40L cells were used to evaluate the agonist activity of this first generation of fusions and to compare it to the CD40L itself. In this assay the level of expression of constructs, their affinity for CD40, and their ability to clusterize them all contributed to the signal. Figure 7A shows that the soluble ectodomain of CD40L alone had a weak CD40 agonist activity as expected. However, the oligomeric SP-D–CD40L fusions had impressive CD40 agonist activities in the same ranking order as the one established by ELISA. The most effective CD40 agonist formats were those with the 12 collagen repeats (12R Col, with and without the linker). To fine tune the optimal collagen domain, a second generation of SP-D–CD40L constructs with different collagen domain sizes and/or sequences were generated and tested in the same assay. None of these second-generation constructions had a better CD40 binding or agonist activity than the 12R Col molecule (Supplementary Figure S7). Interestingly, a molecule with 12 collagen repeats selected in another part of SP-D collagen domain (12bis COL) was clearly not as efficient as the original 12R Col molecule.

The three most promising constructions (i.e., 12R Col, 12R Col linker, and 0R Col linker) were vectorized in vaccinia virus at the TK locus by homologous recombination. The recombinant viruses were used to infect HeLa cells, and the culture medium obtained after 48 h of infection were tested for CD40 agonist activity. The results obtained with samples from recombinant viruses were strikingly different from those obtained with the infection/transfection samples (Figure 7B). Indeed, the VV–0R Col linker produced culture supernatants with much better agonist activity compared to the two VV encoding 12 collagen repeat constructs. The low agonist activity of samples from VV–12R Col-infected cells was due to a lower level of expression of the fusion compared to the VV–0R Col linker (Supplementary Figure S8) or to its corresponding infection/transfection samples (not shown). This result was rather unexpected as usually the vectorization of a given transgene increases its expression level compared to the corresponding infection/transfection. Finally, the VV–0R Col linker had the same replication and oncolytic activity as the benchmark virus (Supplementary Figures S2A, C), indicating that the transgene did not significantly interfere with the viral cycle.

### SP-D–4-1BBL fusion has improved agonist activity compared to 4-1BBL

**FIGURE 8:**
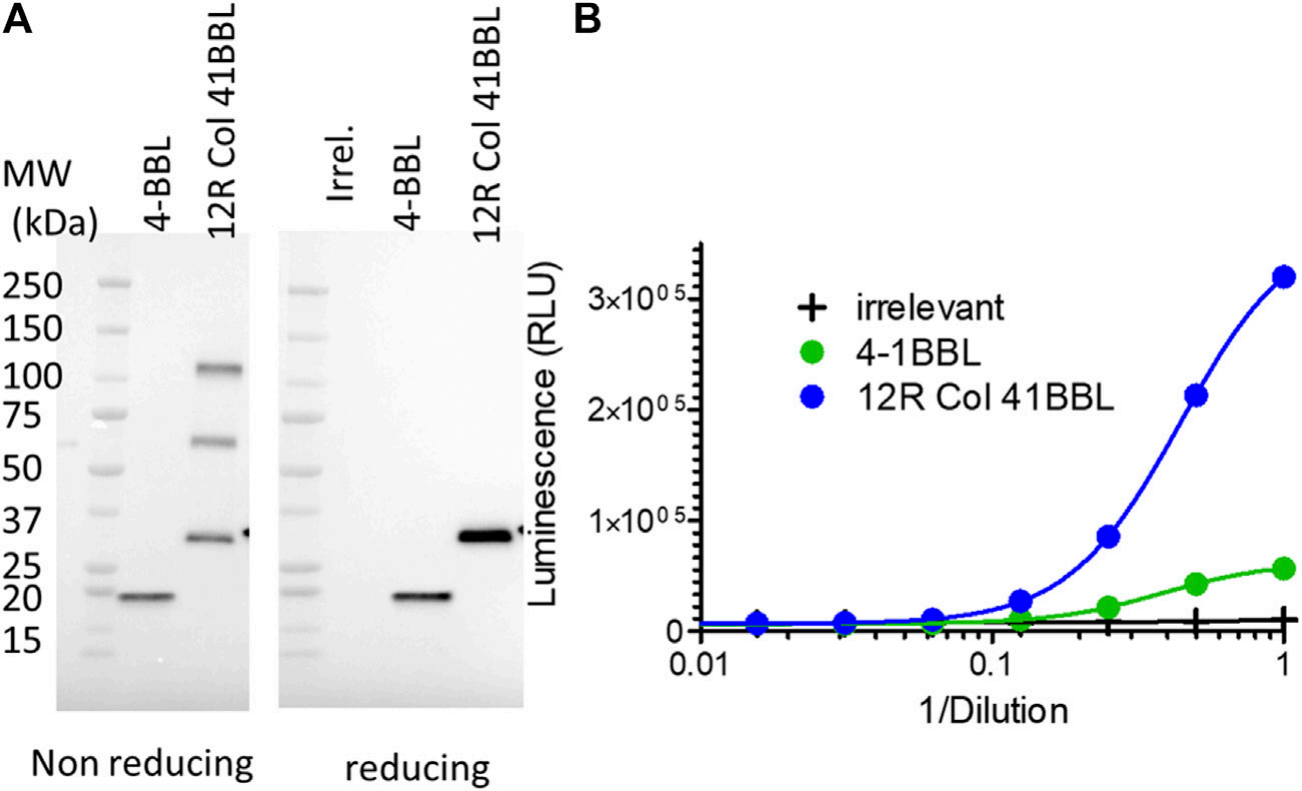
SP-D–4-1BBL fusion is expressed at approximately the same level as 4-1BBL but is a much better 4-1BB agonist. Culture medium of HeLa cells infected/transfected (no recombinant virus was generated) by plasmids encoding SP-D–4-1BBL or –4-1BBL was analyzed for transgene expression by immunoblot using an HRP-conjugated anti-FLAG tag monoclonal antibody **(A)** and their 4-1BB agonist activity using Promega 4-1BB reporter cells **(B)**. 12R COL–4-1BBL is the fusion of oligomeric domains of human SPD containing only 12 collagen repeats and fused to the 4-1BBL ectodomain. 4-1BBL is a construction encoding human CD40L ectodomain. Irr. refers to a plasmid coding an irrelevant protein. For details on constructions, refer to Table 1 and Materials and methods.

The potential of the SP-D oligomeric domain was extended to 4-1BBL as another member of the TNFSF. The CD40L ectodomain was replaced by the 4-1BBL ectodomain in the plasmid encoding the 12R Col construct as the fusion with the best agonist activity in the infection/transfection setting. The two 4-1BBL molecules: 4-1BBL ectodomain alone or fused to the SP-D part of 12R Col (namely, 12R Col 4-1BBL) were expressed at the same level by the infected/transfected cells (Figure 8A). Moreover, the pattern of oligomerization observed on non-reducing conditions was similar for both 12R Col constructs (i.e., CD40L and 4-1BBL fusions). The 4-1BB agonist activity of the 12R Col 4-1BBL construct was clearly superior to 4-1BBL alone (Figure 8B), although the gain of activity was less than the one observed for the equivalent CD40L construct.

## Discussion

PDL1 blockade and/or activation of CD40 or 4-1BB pathways into the TME are proven effective ways to enhance the anti-tumor immune response, particularly in combination with OV treatment (Liu et al., 2017; Lu et al., 2022). While anti-PDL1 treatments are relatively well tolerated, the systemic administration of CD40 or 4-1BB agonists triggers some on-target-off-tumor toxicity which hampers their clinical use. Vectorization of those agonist molecules into the oncolytic vaccinia virus would restrict their expression to the TME since the virus replicates, and then expresses its transgenes, preferentially into tumor cells. Therefore, arming OV with CD40 or 4-1BB agonists would improve the anti-tumoral efficacy of the virus while guaranteeing a subtoxic circulating concentration of those molecules (Kleinpeter et al., 2016; Semmrich et al., 2022).

In this publication, we report the screening of several formats of antagonist anti-PDL1 sdAb as well as CD40 or 4-1BB agonist constructions that were tested in the context of tumor cells infected by an oncolytic vaccinia virus to select the most effective payloads for each pathway.

An anti-PDL1 sdAb was isolated from PDL1-immunized alpacas at the Randox facility using a phage display method. This sdAb was selected for its inhibition of PDL1/PD1 interaction. It was humanized and then vectorized in VV under different formats to optimize its blocking activity. The single-chain dimeric sdAb was found to be the most effective format of the PDL1 blocker once vectorized in vaccinia virus. In addition to its superior blocking activity compared to avelumab, the single-chain dimeric sdAb also has a ∼five-fold smaller size, allowing for potential better diffusion into the tumor (Schmidt and Wittrup, 2009; Li et al., 2016).

Two different approaches were pursued for CD40 or 4-1BB agonist molecules: the first one involved CD40L/4-1BBL ectodomains fused downstream to anti-PDL1 sdAb; the second one used the same ligands fused to the oligomerization domains of SP-D to multimerize them. In both approaches, the tested molecules were secreted to diffuse through the tumor and were devoid of any moiety that increased circulating half-life (such as Fc or serum albumin-binding domains), minimizing their concentrations outside the tumor and any related off-tumor effects. Although each approach was equally effective, they have their own advantages and disadvantages. Unlike the SP-D–TNFSF fusion, the TNFRSF agonist activity of the sdAb fusion depends on a PDL1-positive tumor environment. However, sdAb–TNFSF molecules have a smaller size compared to SP-D constructs and, therefore, theoretically better tumor diffusion. Finally, unlike SP-D–TNFSF, the bispecific sdAb fusion addresses two important targets of the TME by engaging the TNFRSF while blocking the PDL1/PD1 interaction. The expression and TNFSF agonist activities of these different constructions were first assessed on the culture supernatants of HeLa cells infected/transfected with the vaccinia virus and transfer plasmids encoding transgenes under a poxvirus promoter. The viral cycle of vaccinia virus occurs exclusively into the cytoplasm of the infected cells, and viral replication/transcription machineries replicate and transcribe any plasmid into the cytosol of the infected cells as long as it contains a poxvirus promoter (for transcription, see the work of Cochran et al. (1985) and De Silva and Moss (2005)). This property allows the evaluation of numerous payload constructions in the vaccinia virus infection setting without the cumbersome generation of a recombinant virus. This screening allowed the identification of sdAb–linker–TNFSF and SP-D–TNFSF with a collagen domain shortened by ∼80% as the best agonists for each approach. Surprisingly, once vectorized the selected SP-D–CD40L construct was poorly expressed compared to infection/transfection conditions. A version without any collagen domain was found to be much more suitable for vectorization. This difference in expression between infection/transfection and recombinant virus has not previously been observed by the authors with other transgenes, for example, antibodies or antibody fragments (Kleinpeter et al., 2016). This decrease in expression after vectorization could be specific to the presence of the collagen domain. It might also be linked to the fact that in the case of recombinant virus, the expression is localized in a virus factory (*i.e*., a very defined and restricted area of the cytoplasm where the virus replicates (Katsafanas and Moss, 2007)), whereas in the case of infection/transfection, the transcription and translation of transgene occurs across the whole cytoplasm.

Despite this discrepancy between infection and infection/transfection methods, at least one optimized CD40 or 4-1BB agonist and PDL1 blocker have been obtained for vectorization into vaccinia virus.

These results pave the way toward the development of new oncolytic viruses armed with powerful immunomodulators to enhance and sustain the anti-tumoral immune response triggered by infection.

## Data Availability

The original contributions presented in the study are included in the article/Supplementary Material; further inquiries can be directed to the corresponding author.

## References

[B1] Arroyo R. Martin-Gonzalez A. Echaide M. Jain A. Brondyk W. H. Rosenbaum J. (2018). Supramolecular assembly of human pulmonary surfactant protein sp-d. J. Mol. Biol. 430 (10), 1495–1509. 10.1016/j.jmb.2018.03.027 29626540

[B2] Bremer E. (2013). Targeting of the tumor necrosis factor receptor superfamily for cancer immunotherapy. ISRN Oncol. 2013, 1–25. 10.1155/2013/371854 PMC3693168 23840967

[B3] Cochran M. A. Mackett M. Moss B. (1985). Eukaryotic transient expression system dependent on transcription factors and regulatory DNA sequences of vaccinia virus. Proc. Natl. Acad. Sci. U. S. A. 82 (1), 19–23. 10.1073/pnas.82.1.19 3855541 PMC396962

[B4] De Silva F. S. Moss B. (2005). Origin-independent plasmid replication occurs in vaccinia virus cytoplasmic factories and requires all five known poxvirus replication factors. Virol. J. 2, 23. 10.1186/1743-422x-2-23 15784143 PMC1079961

[B5] Fellermeier S. Beha N. Meyer J. E. Ring S. Bader S. Kontermann R. E. (2016). Advancing targeted co-stimulation with antibody-fusion proteins by introducing tnf superfamily members in a single-chain format. Oncoimmunology 5 (11), e1238540. 10.1080/2162402x.2016.1238540 27999756 PMC5139625

[B6] Fend L. Yamazaki T. Remy C. Fahrner C. Gantzer M. Nourtier V. (2017). Immune checkpoint blockade, immunogenic chemotherapy or ifn-alpha blockade boost the local and abscopal effects of oncolytic virotherapy. Cancer Res. 77 (15), 4146–4157. 10.1158/0008-5472.can-16-2165 28536278

[B7] Foloppe J. Kempf J. Futin N. Kintz J. Cordier P. Pichon C. (2019). The enhanced tumor specificity of tg6002, an armed oncolytic vaccinia virus deleted in two genes involved in nucleotide metabolism. Mol. Ther. Oncolytics 14, 1–14. 10.1016/j.omto.2019.03.005 31011628 PMC6461584

[B8] Harmsen M. M. Ruuls R. C. Nijman I. J. Niewold T. A. Frenken L. G. de Geus B. (2000). Llama heavy-chain v regions consist of at least four distinct subfamilies revealing novel sequence features. Mol. Immunol. 37 (10), 579–590. 10.1016/s0161-5890(00)00081-x 11163394

[B9] Haswell L. E. Glennie M. J. Al-Shamkhani A. (2001). Analysis of the oligomeric requirement for signaling by cd40 using soluble multimeric forms of its ligand, cd154. Eur. J. Immunol. 31 (10), 3094–3100. 10.1002/1521-4141(2001010)31:10<3094::aid-immu3094>3.0.co;2-f 11592086

[B10] Kanagavelu S. K. Snarsky V. Termini J. M. Gupta S. Barzee S. Wright J. A. (2012). Soluble multi-trimeric tnf superfamily ligand adjuvants enhance immune responses to a hiv-1 gag DNA vaccine. Vaccine 30 (4), 691–702. 10.1016/j.vaccine.2011.11.088 22146759 PMC3253891

[B11] Katsafanas G. C. Moss B. (2007). Colocalization of transcription and translation within cytoplasmic poxvirus factories coordinates viral expression and subjugates host functions. Cell Host Microbe 2 (4), 221–228. 10.1016/j.chom.2007.08.005 18005740 PMC2084088

[B12] Kleinpeter P. Fend L. Thioudellet C. Geist M. Sfrontato N. Koerper V. (2016). Vectorization in an oncolytic vaccinia virus of an antibody, a fab and a scfv against programmed cell death -1 (pd-1) allows their intratumoral delivery and an improved tumor-growth inhibition. Oncoimmunology 5 (10), e1220467. 10.1080/2162402x.2016.1220467 27853644 PMC5087307

[B13] Kucka K. Wajant H. (2020). Receptor oligomerization and its relevance for signaling by receptors of the tumor necrosis factor receptor superfamily. Front. Cell Dev. Biol. 8, 615141. 10.3389/fcell.2020.615141 33644033 PMC7905041

[B14] Li Z. Krippendorff B. F. Sharma S. Walz A. C. Lave T. Shah D. K. (2016). Influence of molecular size on tissue distribution of antibody fragments. MAbs 8 (1), 113–119. 10.1080/19420862.2015.1111497 26496429 PMC5040103

[B15] Liu Z. Ravindranathan R. Kalinski P. Guo Z. S. Bartlett D. L. (2017). Rational combination of oncolytic vaccinia virus and pd-l1 blockade works synergistically to enhance therapeutic efficacy. Nat. Commun. 8, 14754. 10.1038/ncomms14754 28345650 PMC5378974

[B16] Lu S. C. Hansen M. J. Hemsath J. R. Parrett B. J. Zell B. N. Barry M. A. (2022). Modulating oncolytic adenovirus immunotherapy by driving two axes of the immune system by expressing 4-1bbl and cd40l. Hum. Gene Ther. 33 (5-6), 250–261. 10.1089/hum.2021.197 34731019 PMC11981553

[B17] Maass D. R. Sepulveda J. Pernthaner A. Shoemaker C. B. (2007). Alpaca (lama pacos) as a convenient source of recombinant camelid heavy chain antibodies (vhhs). J. Immunol. Methods 324 (1-2), 13–25. 10.1016/j.jim.2007.04.008 17568607 PMC2014515

[B18] Medler J. Kucka K. Melo V. Zhang T. von Rotenhan S. Ulrich J. (2022). Cd40-and 41bb-specific antibody fusion proteins with pdl1 blockade-restricted agonism. Theranostics 12 (4), 1486–1499. 10.7150/thno.66119 35198053 PMC8825603

[B19] Muller D. (2023). Targeting co-stimulatory receptors of the tnf superfamily for cancer immunotherapy. BioDrugs 37 (1), 21–33. 10.1007/s40259-022-00573-3 36571696 PMC9836981

[B20] Pandey M. S. Wang C. Umlauf S. Lin S. (2021). Simultaneous inhibition of pd-1 and stimulation of cd40 signaling pathways by anti-pd-l1/cd40l bispecific fusion protein synergistically activate target and effector cells. Int. J. Mol. Sci. 22 (21), 11302. 10.3390/ijms222111302 34768776 PMC8583728

[B21] Schmidt M. M. Wittrup K. D. (2009). A modeling analysis of the effects of molecular size and binding affinity on tumor targeting. Mol. Cancer Ther. 8 (10), 2861–2871. 10.1158/1535-7163.mct-09-0195 19825804 PMC4078872

[B22] Semmrich M. Marchand J. B. Fend L. Rehn M. Remy C. Holmkvist P. (2022). Vectorized Treg-depleting αCTLA-4 elicits antigen cross-presentation and CD8^+^ T cell immunity to reject ‘cold’ tumors. J. Immunother. Cancer 10 (1), e003488. 10.1136/jitc-2021-003488 35058324 PMC8783833

[B23] Stone G. W. Barzee S. Snarsky V. Kee K. Spina C. A. Yu X. F. (2006). Multimeric soluble cd40 ligand and gitr ligand as adjuvants for human immunodeficiency virus DNA vaccines. J. Virol. 80 (4), 1762–1772. 10.1128/jvi.80.4.1762-1772.2006 16439533 PMC1367159

[B24] Wan P. K. Ryan A. J. Seymour L. W. (2021). Beyond cancer cells: targeting the tumor microenvironment with gene therapy and armed oncolytic virus. Mol. Ther. 29 (5), 1668–1682. 10.1016/j.ymthe.2021.04.015 33845199 PMC8116634

[B25] White M. Kingma P. Tecle T. Kacak N. Linders B. Heuser J. (2008). Multimerization of surfactant protein d, but not its collagen domain, is required for antiviral and opsonic activities related to influenza virus. J. Immunol. 181 (11), 7936–7943. 10.4049/jimmunol.181.11.7936 19017984

[B26] Wyzgol A. Muller N. Fick A. Munkel S. Grigoleit G. U. Pfizenmaier K. (2009). Trimer stabilization, oligomerization, and antibody-mediated cell surface immobilization improve the activity of soluble trimers of cd27l, cd40l, 41bbl, and glucocorticoid-induced tnf receptor ligand. J. Immunol. 183 (3), 1851–1861. 10.4049/jimmunol.0802597 19596991

[B27] Zhai T. Wang C. Xu Y. Huang W. Yuan Z. Wang T. (2021). Generation of a safe and efficacious llama single-domain antibody fragment (vhh) targeting the membrane-proximal region of 4-1bb for engineering therapeutic bispecific antibodies for cancer. J. Immunother. Cancer 9 (6), e002131. 10.1136/jitc-2020-002131 34172514 PMC8237747

