# Supplementary material for: Design and selection of anti-PD-L1 single-domain antibody and tumor necrosis factor superfamily ligands for an optimal vectorization in an oncolytic virus
Source: Front Bioeng Biotechnol. 2023 Nov 15;11:1247802. doi: 10.3389/fbioe.2023.1247802 (PMC10694795; doi:10.3389/fbioe.2023.1247802)
Supplement: Supplementary file 1 [file Presentation1.PPTX]

## Slide 1
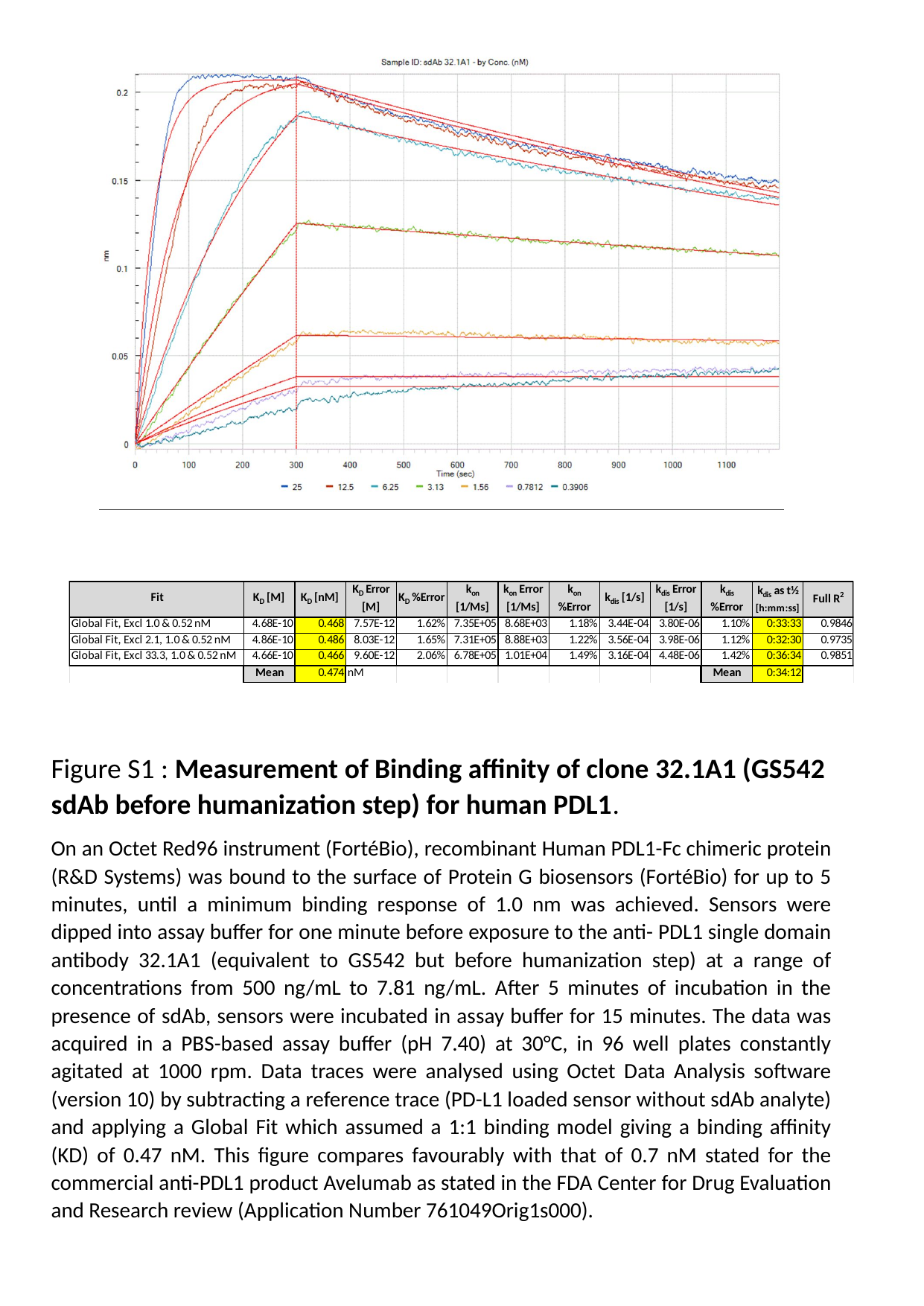

Figure S1 : Measurement of Binding affinity of clone 32.1A1 (GS542 sdAb before humanization step) for human PDL1.
On an Octet Red96 instrument (FortéBio), recombinant Human PDL1-Fc chimeric protein (R&D Systems) was bound to the surface of Protein G biosensors (FortéBio) for up to 5 minutes, until a minimum binding response of 1.0 nm was achieved. Sensors were dipped into assay buffer for one minute before exposure to the anti- PDL1 single domain antibody 32.1A1 (equivalent to GS542 but before humanization step) at a range of concentrations from 500 ng/mL to 7.81 ng/mL. After 5 minutes of incubation in the presence of sdAb, sensors were incubated in assay buffer for 15 minutes. The data was acquired in a PBS-based assay buffer (pH 7.40) at 30°C, in 96 well plates constantly agitated at 1000 rpm. Data traces were analysed using Octet Data Analysis software (version 10) by subtracting a reference trace (PD-L1 loaded sensor without sdAb analyte) and applying a Global Fit which assumed a 1:1 binding model giving a binding affinity (KD) of 0.47 nM. This figure compares favourably with that of 0.7 nM stated for the commercial anti-PDL1 product Avelumab as stated in the FDA Center for Drug Evaluation and Research review (Application Number 761049Orig1s000).

## Slide 2
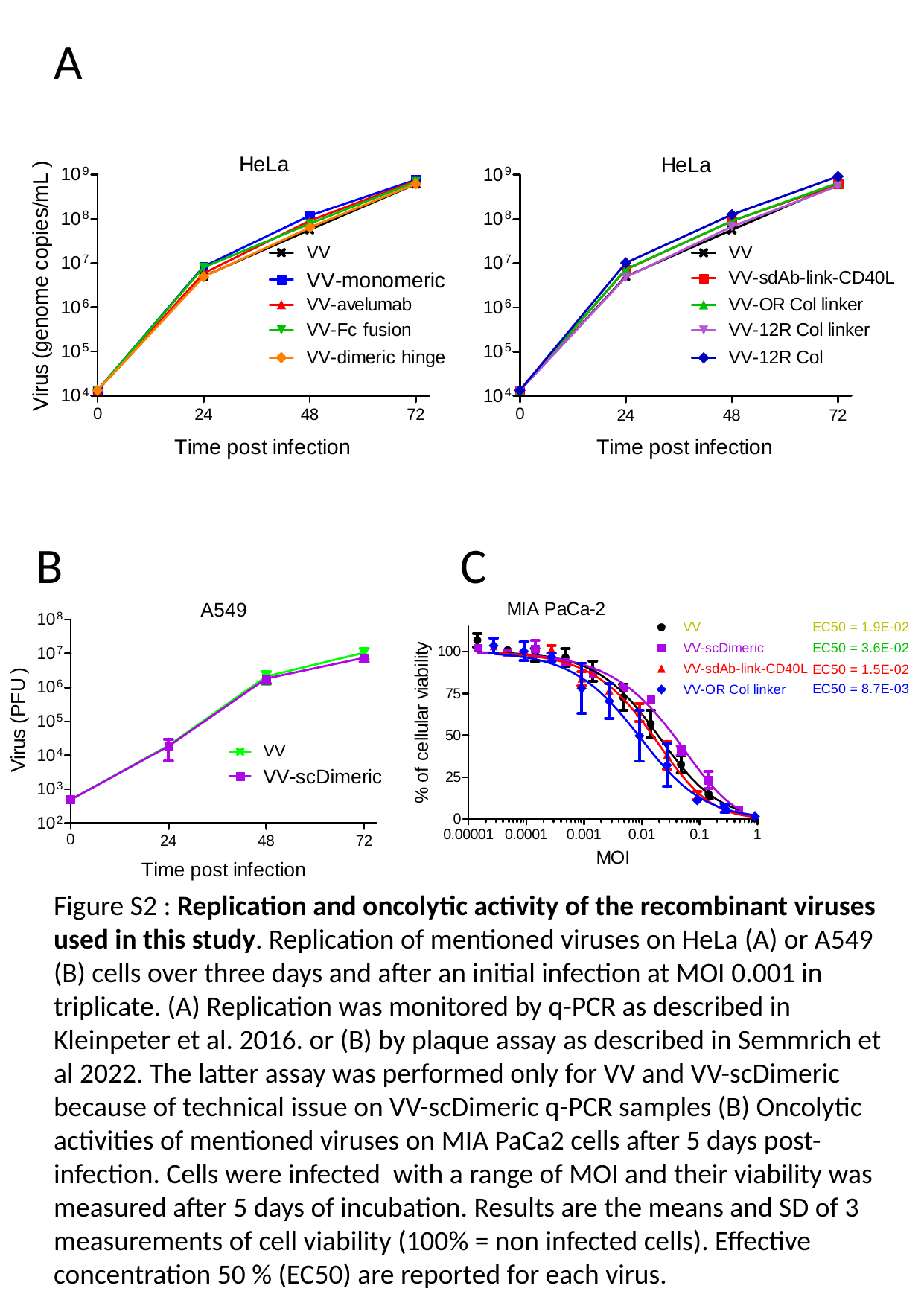

A
B
C
Figure S2 : Replication and oncolytic activity of the recombinant viruses used in this study. Replication of mentioned viruses on HeLa (A) or A549 (B) cells over three days and after an initial infection at MOI 0.001 in triplicate. (A) Replication was monitored by q-PCR as described in Kleinpeter et al. 2016. or (B) by plaque assay as described in Semmrich et al 2022. The latter assay was performed only for VV and VV-scDimeric because of technical issue on VV-scDimeric q-PCR samples (B) Oncolytic activities of mentioned viruses on MIA PaCa2 cells after 5 days post-infection. Cells were infected with a range of MOI and their viability was measured after 5 days of incubation. Results are the means and SD of 3 measurements of cell viability (100% = non infected cells). Effective concentration 50 % (EC50) are reported for each virus.

## Slide 3
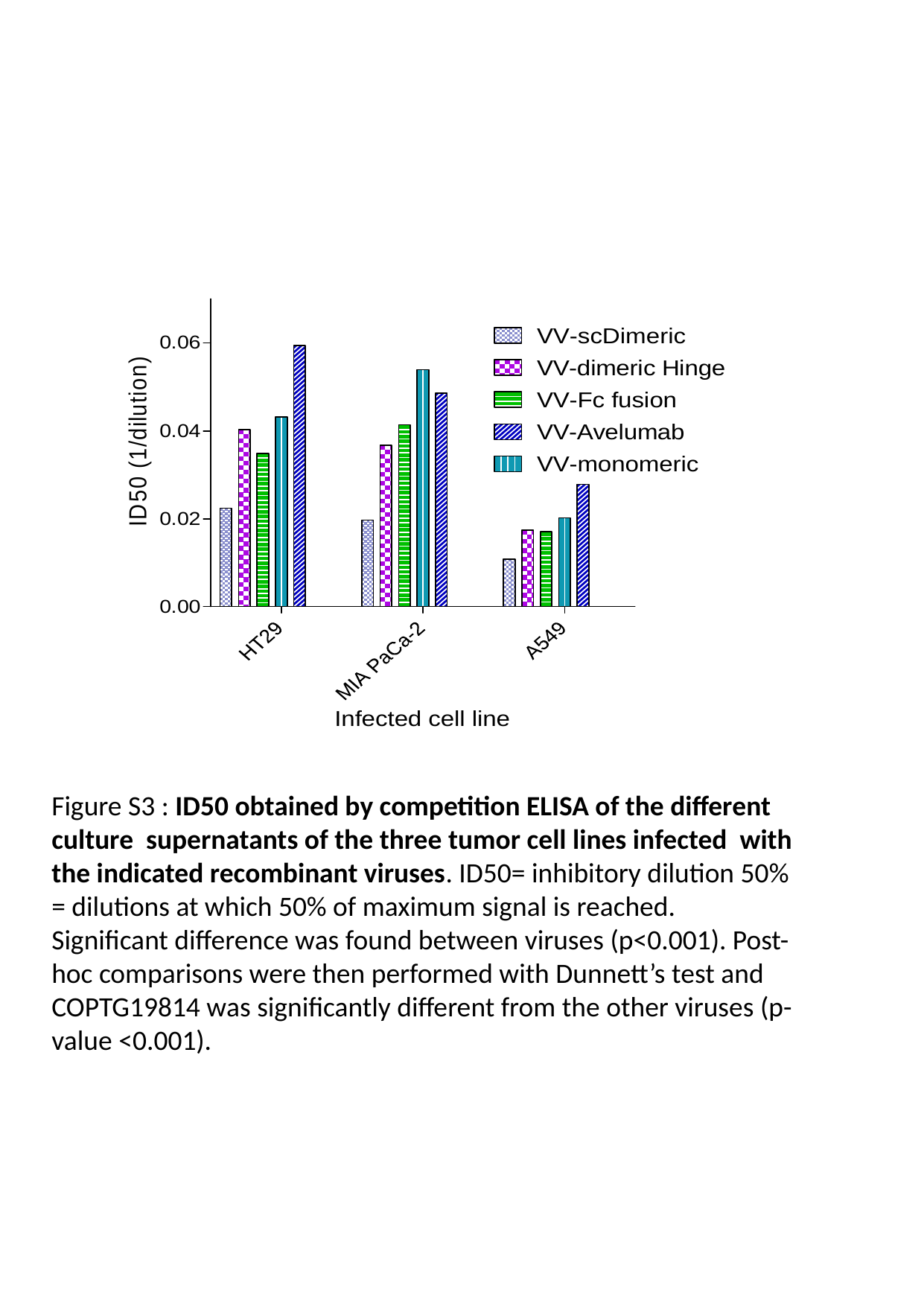

Figure S3 : ID50 obtained by competition ELISA of the different culture supernatants of the three tumor cell lines infected with the indicated recombinant viruses. ID50= inhibitory dilution 50% = dilutions at which 50% of maximum signal is reached. Significant difference was found between viruses (p<0.001). Post-hoc comparisons were then performed with Dunnett’s test and COPTG19814 was significantly different from the other viruses (p-value <0.001).

## Slide 4
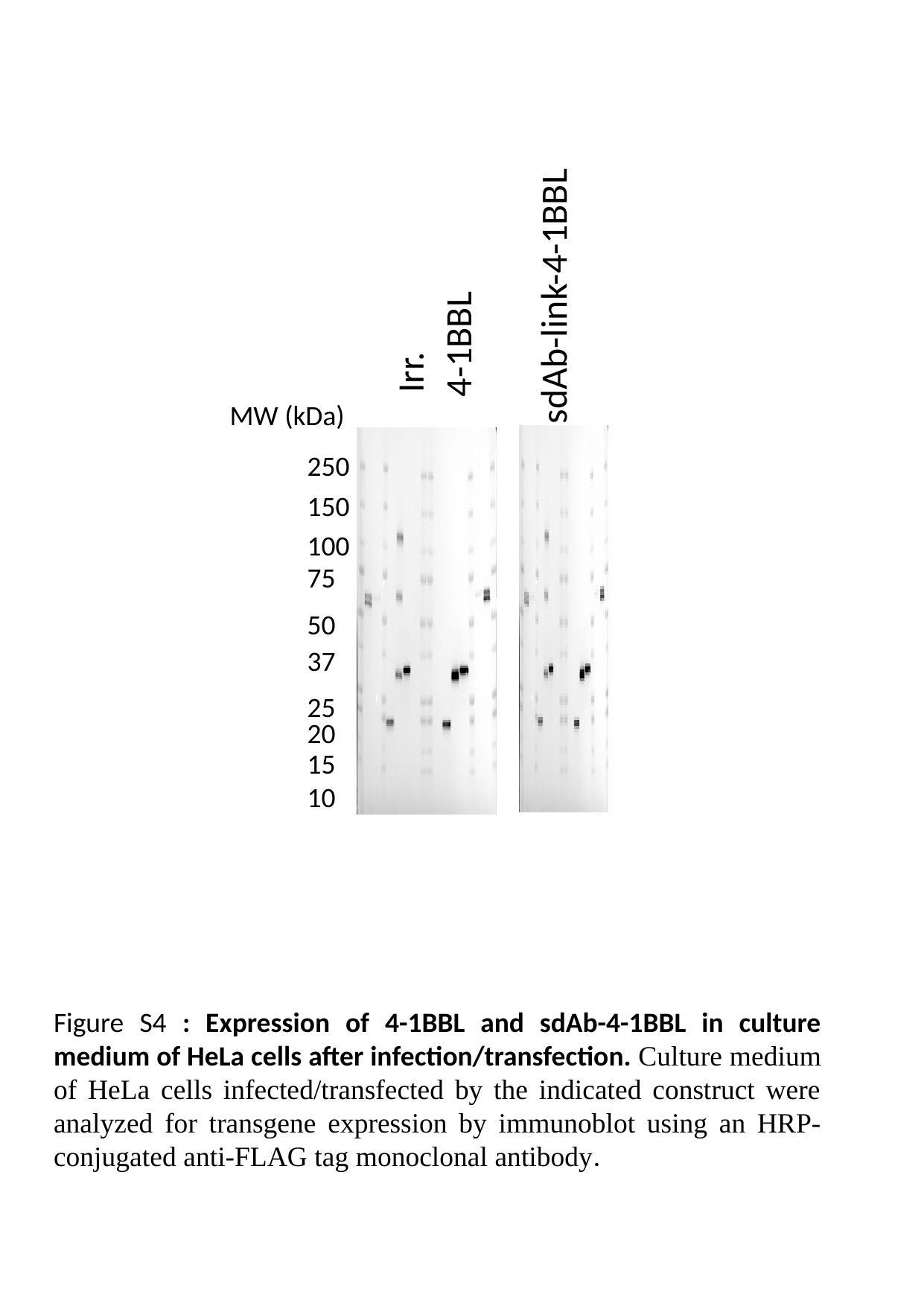

sdAb-link-4-1BBL
4-1BBL
Irr.
MW (kDa)
250
150
100
75
50
37
25
20
15
10
Figure S4 : Expression of 4-1BBL and sdAb-4-1BBL in culture medium of HeLa cells after infection/transfection. Culture medium of HeLa cells infected/transfected by the indicated construct were analyzed for transgene expression by immunoblot using an HRP-conjugated anti-FLAG tag monoclonal antibody.

## Slide 5
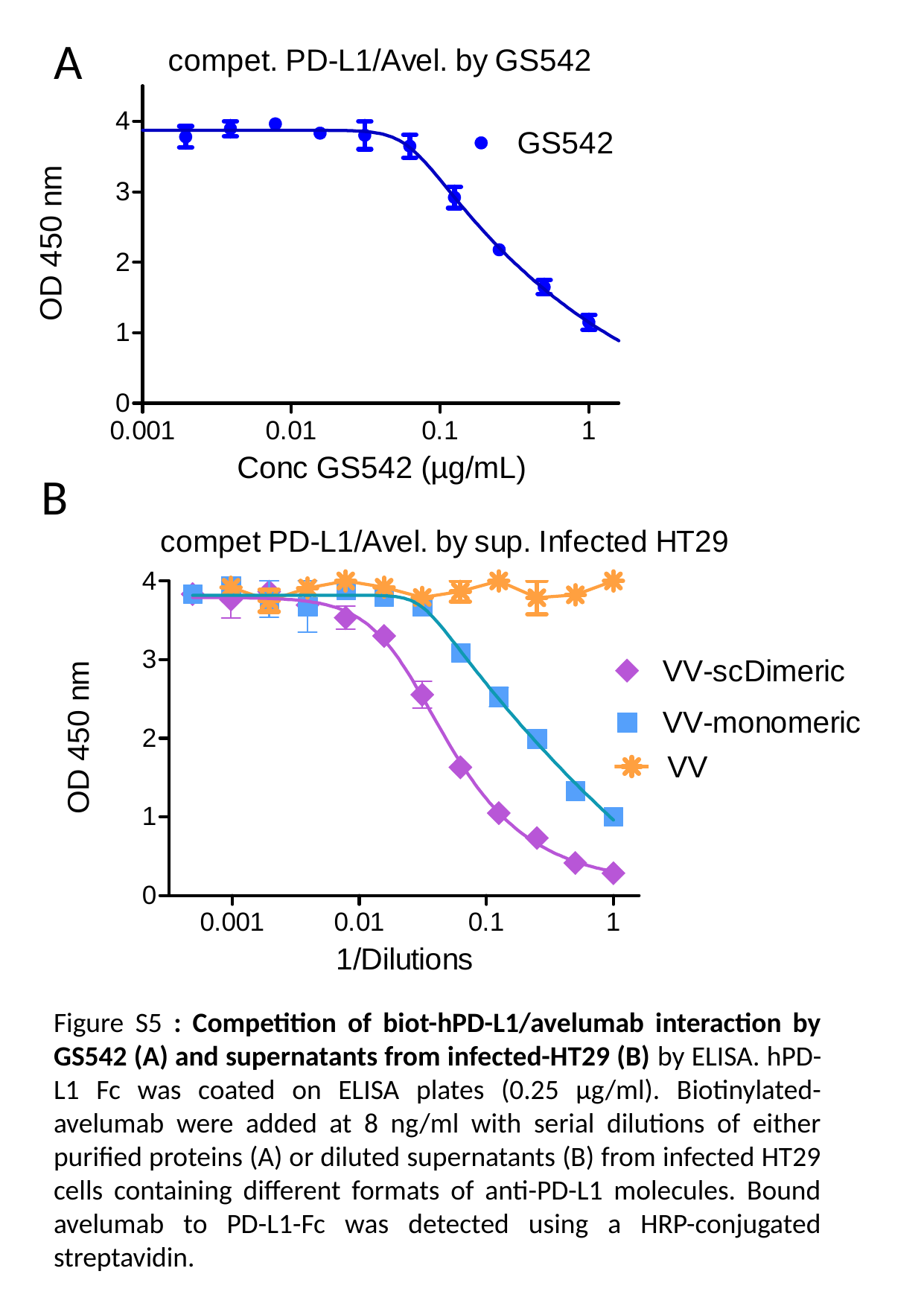

A
B
Figure S5 : Competition of biot-hPD-L1/avelumab interaction by GS542 (A) and supernatants from infected-HT29 (B) by ELISA. hPD-L1 Fc was coated on ELISA plates (0.25 µg/ml). Biotinylated- avelumab were added at 8 ng/ml with serial dilutions of either purified proteins (A) or diluted supernatants (B) from infected HT29 cells containing different formats of anti-PD-L1 molecules. Bound avelumab to PD-L1-Fc was detected using a HRP-conjugated streptavidin.

## Slide 6
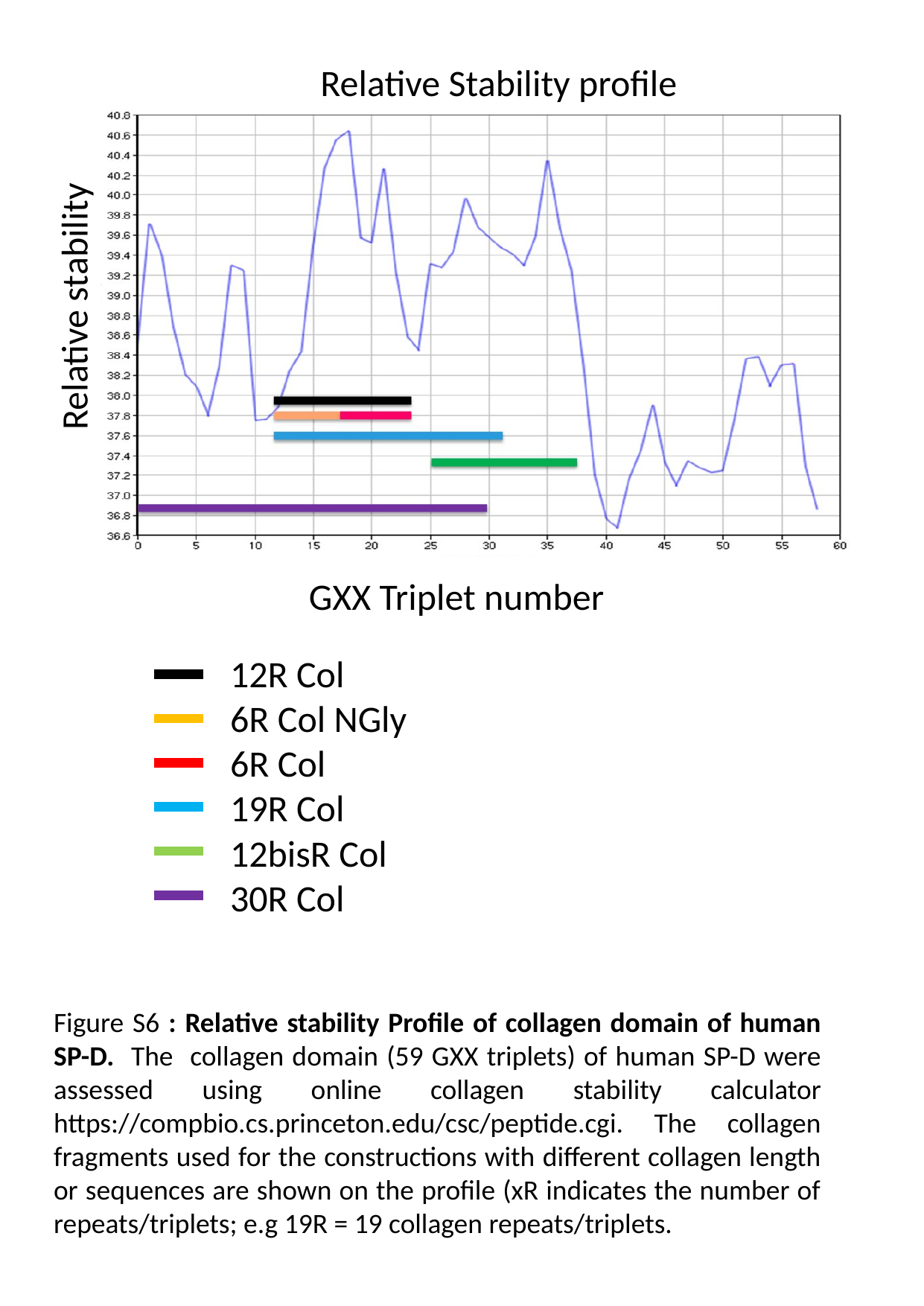

Relative Stability profile
Relative stability
GXX Triplet number
12R Col
6R Col NGly
6R Col
19R Col
12bisR Col
30R Col
Figure S6 : Relative stability Profile of collagen domain of human SP-D. The collagen domain (59 GXX triplets) of human SP-D were assessed using online collagen stability calculator https://compbio.cs.princeton.edu/csc/peptide.cgi. The collagen fragments used for the constructions with different collagen length or sequences are shown on the profile (xR indicates the number of repeats/triplets; e.g 19R = 19 collagen repeats/triplets.

## Slide 7
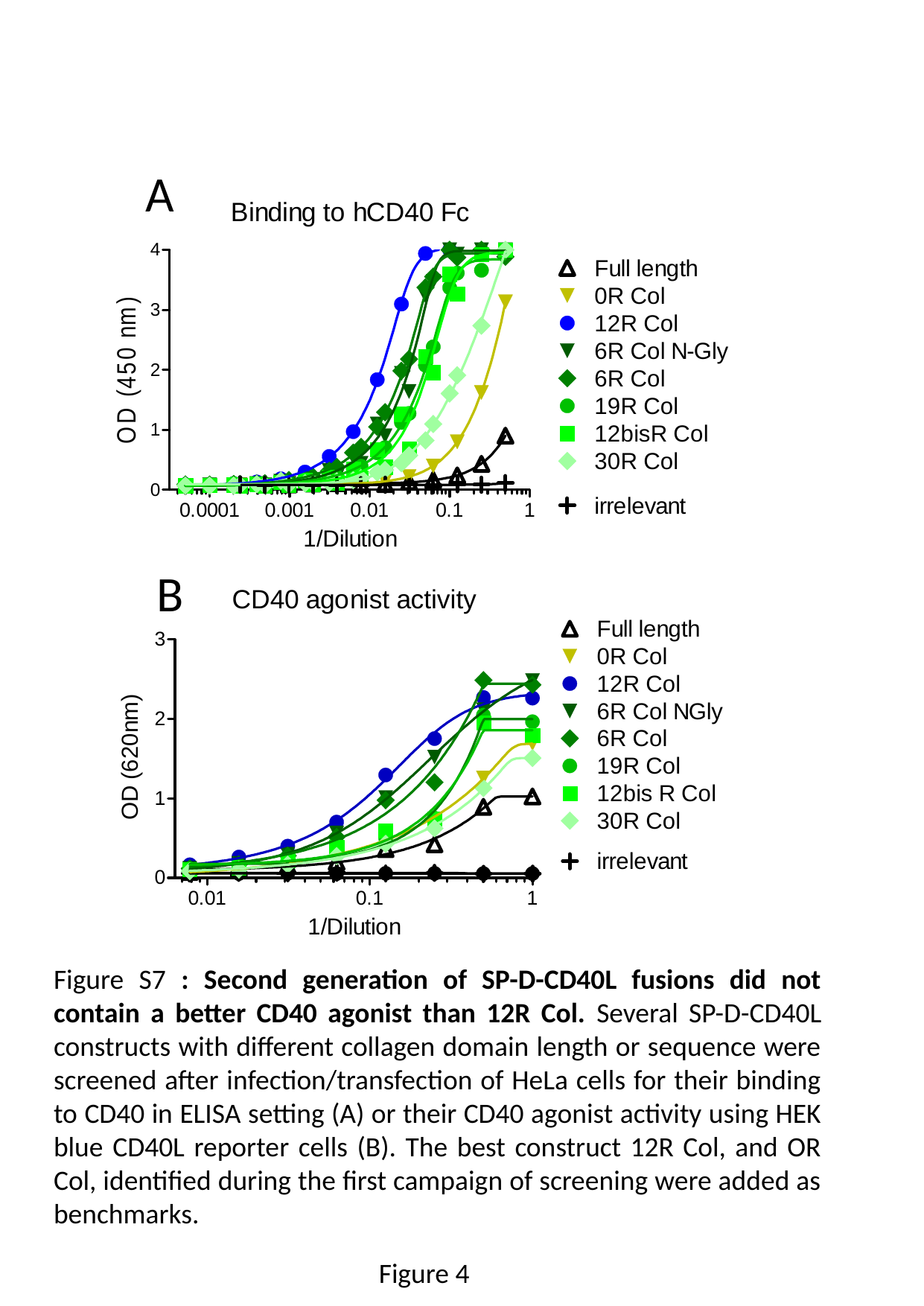

A
B
Figure S7 : Second generation of SP-D-CD40L fusions did not contain a better CD40 agonist than 12R Col. Several SP-D-CD40L constructs with different collagen domain length or sequence were screened after infection/transfection of HeLa cells for their binding to CD40 in ELISA setting (A) or their CD40 agonist activity using HEK blue CD40L reporter cells (B). The best construct 12R Col, and OR Col, identified during the first campaign of screening were added as benchmarks.
Figure 4

## Slide 8
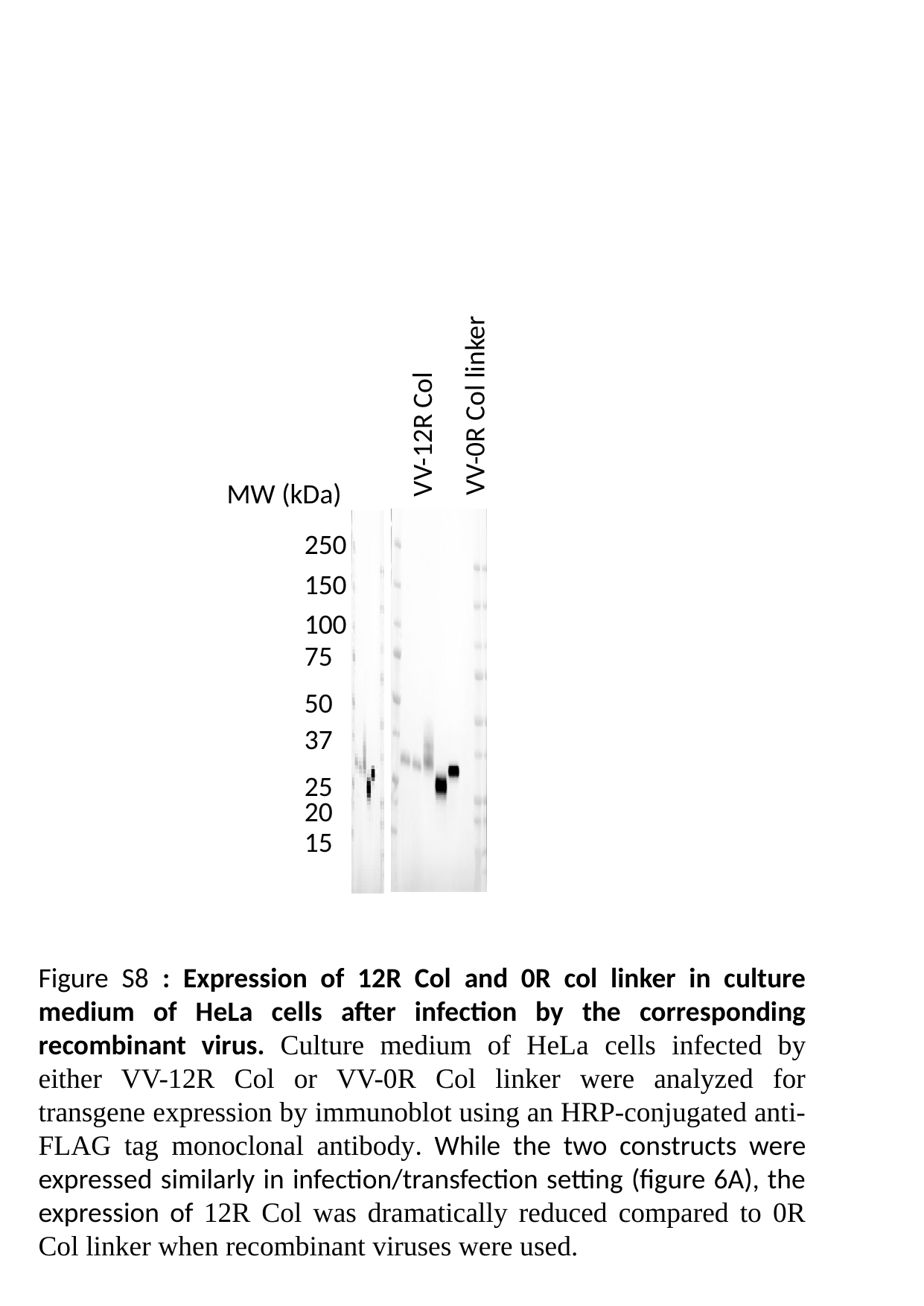

VV-0R Col linker
VV-12R Col
MW (kDa)
250
150
100
75
50
37
25
20
15
Figure S8 : Expression of 12R Col and 0R col linker in culture medium of HeLa cells after infection by the corresponding recombinant virus. Culture medium of HeLa cells infected by either VV-12R Col or VV-0R Col linker were analyzed for transgene expression by immunoblot using an HRP-conjugated anti-FLAG tag monoclonal antibody. While the two constructs were expressed similarly in infection/transfection setting (figure 6A), the expression of 12R Col was dramatically reduced compared to 0R Col linker when recombinant viruses were used.
